# What is the Evidence on Lifestyle Interventions for the Symptom Management of Pelvic Pain in Women With Endometriosis or Adenomyosis? A Scoping Review

**DOI:** 10.1177/15598276261419770

**Published:** 2026-02-24

**Authors:** Bethany Hough, Natalie Drever, Sam Manger

**Affiliations:** 1 Department of Medicine and Dentistry, 8001 James Cook University, Townsville, QLD, Australia (BH, ND, SM); 2 Department of Obstetrics & Gynaecology, Cairns Hospital, QLD, Cairns, Australia (ND)

**Keywords:** endometriosis, adenomyosis, pelvic pain, lifestyle intervention, nutrition, physical activity, mind-body

## Abstract

Endometriosis and adenomyosis are chronic, debilitating, inflammatory conditions affecting women of reproductive age. Current management primarily focuses on pharmacologic therapies and surgical interventions; however, many individuals adopt self-directed lifestyle modifications despite limited evidence-based guidance to support these approaches. This scoping review aimed to collate and evaluate the available evidence for lifestyle interventions in the management of pelvic pain among women with endometriosis and/or adenomyosis, with consideration of key lifestyle medicine domains including nutrition, physical activity, mind–body practices, social connection, sleep, substance cessation, and self-management. The review was conducted in accordance with the Preferred Reporting Items for Systematic Reviews and Meta-Analyses extension for Scoping Reviews (PRISMA-ScR). Electronic databases (MEDLINE [OVID], CINAHL, and Scopus) were systematically searched to identify relevant interventional studies. Following abstract and full-text screening, 21 studies met inclusion criteria. Included studies demonstrated substantial heterogeneity in methodology, sample size, intervention type, and duration of follow-up. Only one study specifically included participants with adenomyosis. Findings suggested potential benefits of dietary modification, physical activity, mindfulness-based interventions, yoga, digital health programs, and transcutaneous electrical nerve stimulation (TENS) for pelvic pain management. However, small sample sizes and methodological variability limit the strength of conclusions. Overall, the current evidence base remains limited and underscores the need for rigorously designed interventional studies. Future research should incorporate broader outcome domains, including quality of life, fertility, and mental health, and should more comprehensively address lifestyle domains such as sleep and substance use. Greater inclusion of individuals with adenomyosis is also essential to inform evidence-based lifestyle recommendations in this population.

“Hormonal treatments aim to slow or suppress the growth of endometriosis lesions or stop bleeding and thus reduce the pain and severity of endometriosis”.

## Introduction

Endometriosis is a common, chronic, debilitating women’s health condition which impacts at least 1 in 9 Australian women by the age of 44.^1^ It is a chronic inflammatory condition diagnosed by the presence of endometrial-like tissue outside of the uterus.^2^ The endometrial-like tissue can occur in the pelvis (such as superficial peritoneum and ovaries), as well as extra-pelvic sites including the abdominal organs, lungs and nervous system with sites and severity varying from person-to-person. This results in various symptoms in sufferers including period pain (dysmenorrhoea), heavy menstrual bleeding (menorrhagia), chronic pelvic pain, painful sex (dyspareunia), painful defecation (dyschezia), fatigue and infertility.^3^ Symptoms vary between women and the degree of disease does not always correlate to symptom severity.^3^ These symptoms often negatively impact daily living and quality of life.^4^

Adenomyosis often co-exists with endometriosis and occurs when the endometrium grows into the muscular wall of the uterus (myometrium). It can also cause debilitating symptoms including pelvic pain, dysmenorrhoea, menorrhagia, infertility and poor pregnancy outcomes.^5^ Prevalence estimates of adenomyosis vary widely from 8.8% to 61.5%.^6^

Previously, the ‘gold standard’ for diagnosis of endometriosis was through laparoscopy and biopsy of suspicious lesions with histological confirmation of endometriosis, however there is a growing role for the use of specialised ultrasound (US) and magnetic resonance imaging (MRI) in the diagnosis and evaluation of the extent of disease.^7^ Despite increasing awareness and improved diagnostic techniques, there is an average delay of 6.4 years from symptom onset to diagnosis of endometriosis in Australian women.^8^ Historically, adenomyosis was a histopathologic diagnosis after hysterectomy, however due to imaging advancements, it can now be diagnosed by non-invasive techniques such as US and MRI.^5^

Current endometriosis management is focused on analgesia to manage pain (with non-steroidal anti-inflammatories (NSAIDs) being first line), hormonal medical treatments to regulate oestrogen, laparoscopic surgery for excision of extra endometrial tissue or a combination of all three.^9^ Importantly, it is unclear whether excision of endometriosis reduces pain or improves quality of life when compared to diagnostic or placebo/sham laparoscopy only.^10^ Even with complete excision of all endometriosis lesions in the pelvis, pain recurrence at 5 years is approximately 50%.^11^ In some cases, hysterectomy is also performed. Hormonal treatments aim to slow or suppress the growth of endometriosis lesions or stop bleeding and thus reduce the pain and severity of endometriosis. Hormonal treatment options include the combined oral contraceptive pill (COCP), progesterone (oral form, subcutaneous implant or intrauterine device) or gonadotrophin-releasing hormone agonists and antagonists.^9^ Reported discontinuation rates of COCP and progesterone hormonal treatment is reportedly up to 50% owing to unwanted side effects (Brown et al., 2014).

Adenomyosis has a similar treatment approach with medical options to help with pain and menorrhagia (including NSAIDs, tranexamic acid and hormonal options as listed above) or surgical options (including endometrial ablation, excision of adenomyosis and hysterectomy).^12^ Uterine artery embolisation may also be performed in adenomyosis.^12^

Previous Royal Australian and New Zealand College of Obstetricians and Gynaecologists (RANZCOG) endometriosis and adenomyosis guidelines published in 2021 only contained a brief segment on non-pharmacological and non-surgical recommendations for management of pain associated with these conditions. The recommendations included are low evidence for the role of Chinese herbal medicines and very low to moderate evidence for the role of acupuncture in endometriosis. The guidelines report there is little to no evidence for non-pharmacological interventions in the management of adenomyosis.^9^

Women with endometriosis are often unhappy with their current health care, with a 2018 study illustrating that only 54.6% of women were satisfied with the medical care received.^13^ Many women take their health care into their own hands, with an online Australian survey demonstrating 76% of those with endometriosis currently utilising self-care or lifestyle choices to manage their condition.^14^ These women report improved pain outcomes with a mean self-reported effectiveness in pain reduction of 6.52 points for heat therapy and 6.39 points for dietary changes on a 10-point scale.^14^

Lifestyle medicine is a growing field of multi-disciplinary health care which is defined as ‘the application of environmental, behavioural and motivational principles including self-care and self-management, to the management of lifestyle-related health problems in a clinical setting’.^15^ It is often considered in broad pillars including nutrition, physical activity, mind-body, sleep, social connection and substance cessation. Lifestyle interventions are effective in reducing systemic inflammation.^16-18^ There is emerging research indicating potential benefit for these interventions in chronic disabling inflammatory conditions such as rheumatoid arthritis or inflammatory bowel disease.^19,20^

A systematic review and meta-analysis in 2024 examined the effect of dietary intervention on endometriosis.^21^ It analysed 11 randomised control trials; however, the studies included all focused on dietary supplementation and did not include analysis of non-supplement based dietary interventions. There was suggested evidence for the role of vitamins C and E, fish oil (omega 3/6), wobenzym vital (an enzyme supplement containing the enzymes bromelain, papain, trypsin & chymotrypsin, bioflavonoids and vitamins C, D and E) and garlic supplementation for dysmenorrhoea. There was mixed evidence for vitamin D; however, the overall quality of included studies was poor with high heterogeneity and risk of bias limiting the clinical applicability.^21^ Given this review has already taken place, and supplementation is not traditionally included in lifestyle medicine, further analysis of the role of dietary supplementation in endometriosis and adenomyosis will not be included in this review.

Despite the high prevalence of women with endometriosis utilising self-care strategies and lifestyle changes to optimise their management, there have been no previous reviews identified on the lifestyle management of endometriosis and adenomyosis. This scoping review aims to assess the current available state of evidence for lifestyle interventions in endometriosis and adenomyosis analysing the impact of diet, physical activity, social connection, sleep, mind-body interventions, substance cessation and self-care strategies on pelvic pain management.

## Methodology

Scoping reviews are a research methodology which can be utilised to meet several objectives including; analysis of the extent of evidence on a topic, summarising findings from a body of knowledge and identifying gaps in the literature to aid planning of future research.^22^ Given the non-pharmacological and lifestyle management of endometriosis and adenomyosis is a poorly researched area, a scoping review was considered the most appropriate methodology to map the current restricted evidence and thus answer the research question of ‘What is the evidence of lifestyle interventions for the symptom management of pelvic pain in women with endometriosis or adenomyosis?’.

Guidelines in the Preferred Reporting Items for Systematic Reviews and Meta-Analysis extension for Scoping Reviews (PRISMA-ScR) were followed during this review.^23^

To identify relevant studies, the following bibliography databases of Medline (OVID), Cumulated Index in Nursing and Allied Health Literature (CINAHL) and Scopus were utilised. The search strategy was drafted by author BJ with guidance from experienced librarian, Stephen Anderson, and further refined through team discussion. A combination of MeSH subject headings and keyword searches was used in Medline and CINAHL, and keyword searches were used in Scopus utilising a **P**opulation, **I**ntervention, **C**omparison(s), **O**utcome (PICO) approach. This PICO approach was thought to be the most appropriate to address the clinical question.^24^

Patient/population – women with endometriosis and/or adenomyosis.

Intervention – lifestyle intervention.

Comparison – no lifestyle intervention (either with comparison of a control group or pre-intervention)

Outcome – Pelvic Pain

When reflecting on lifestyle interventions, each lifestyle medicine pillar was considered individually to ensure a search strategy which covered the breadth of lifestyle interventions. Self-management and self-care key words were also included to cover lifestyle interventions not categorised in the traditional pillar model. Synonyms of pelvic pain, such as chronic pelvic pain, dysmenorrhoea and dyspareunia, were included.

Appendix A contains a table of the complete search strategy.

### Inclusion Criteria/Exclusion Criteria

Following the PICO approach, inclusion and exclusion criteria were developed. The population was limited to human studies of women with endometriosis and/or adenomyosis. To address the research question, any intervention which focused on one or more of the pillars of lifestyle medicine, or another self-management strategy, and the impact of this intervention on pelvic pain were included.

Any intervention which required long-term direct allied health/specialist involvement and could not be self-managed long-term were excluded. This included interventions such as acupuncture or massage. Interventions which required allied health input initially for education but could then be self-managed were included (such as psychology in mindfulness education, physiotherapy in a physical activity program or dietician in a dietary intervention). Dietary interventions had to be wholly diet based and could not involve supplementations.

Given primary searches demonstrated limited randomised controlled trials in this area, the decision was made to include all interventional trails. This also includes interventional trials with a control group that are not randomised, along with intervention trials which do not have a control group, but instead, compared the research group to themselves pre and post intervention. Any non-interventional studies, review articles, editorials, letters, conference proceedings, poster presentations, commentaries or protocols were excluded.

Given the review is aiming to map the scope of the topic, a publish date limit was not included.^22^ Table 1 below contains the inclusion and exclusion criteria.

**Table 1.** Inclusion & Exclusion Criteria.

Inclusion	Exclusion
English language	Non-English language
Women over the age of 18 with a diagnosis of endometriosis or adenomyosis (on histology or imaging)	Animal studies
	Women without confirmed endometriosis or adenomyosis
	Women under the age of 18
Studies on interventions which focus on any of the pillars of lifestyle medicine or self-management strategies and explores the impact of these interventions on pelvic pain management	Studies that do not explore the impact of the lifestyle intervention on pelvic pain management
Studies on interventions which can be self-managed and do not require direct ongoing input from a medical specialist or allied health practitioner after initiation	Studies on interventions which required direct ongoing input from medical specialist or allied health practitioners after initiation
	Studies focused on the impact of nutritional supplementation
Original interventional research	Secondary review studies, editorials, letters, conference proceedings, poster presentations, commentaries, protocols, non-interventional studies
Full text, able to access by institution	Non-full text (i.e. abstract only), unable to access by institution

## Results

### Study Selection

The complete search took place on Medline (OVID), CINHAL and Scopus on the 31^st^ of March 2025. The search from Medline (OVID) returned 482 references, CINHAL returned 66 references and Scopus returned 3653 references. These references were all uploaded onto Covidence with a total of 294 duplicates removed. The remaining 3907 articles underwent title and abstract screening by two reviewers independently (authors BH and ND) against the inclusion and exclusion criteria. Disagreements were noted and discussed between the reviewers, and 25 articles were included for the full text screening. Six articles were excluded following this full text screening with 19 articles included in the final review. Three of these articles were excluded due to wrong outcomes, two due to wrong study design and one as it was a protocol and the interventional trial had not been completed. Two additional articles were added from reference searching.

**Figure 1.**
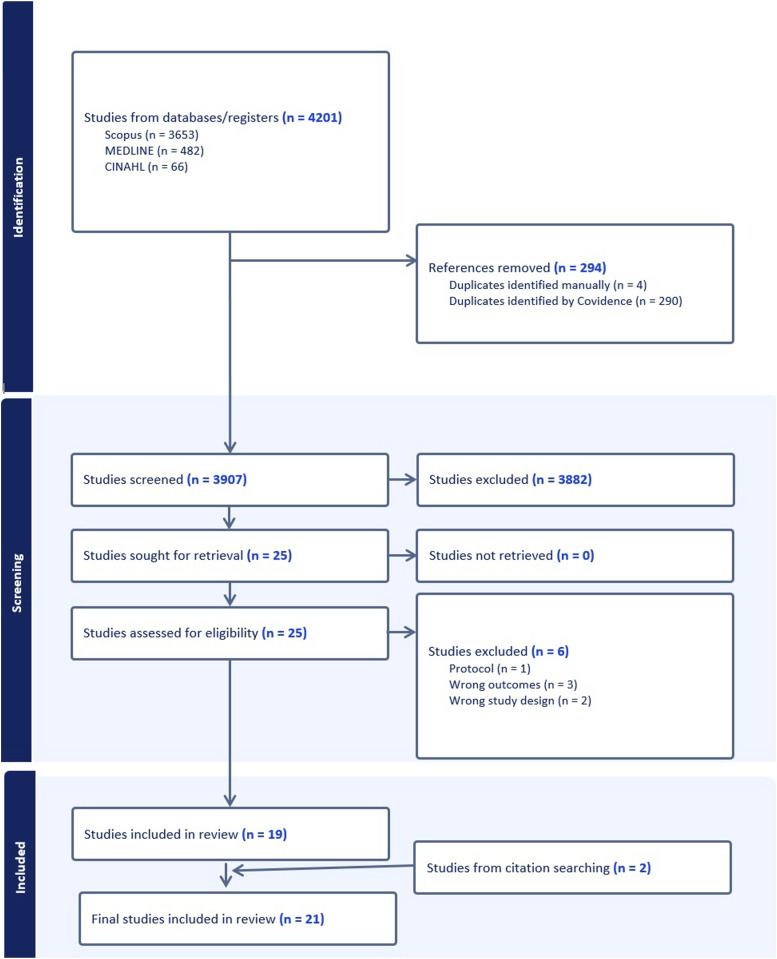
PRISMA chart showing selection of studies for the scoping review

Given that a scoping review aims to understand the extent of the literature rather than synthesise the data, a risk of bias was not considered in study selection.^23^ Figure 1 below is a PRISMA chart illustrating the selection of studies for the scoping review.

### Data Extraction, Charting and Synthesis

Data extraction was carried out using a data charting form by one author (BH). The completed data charting form is attached in Appendix B.

**Figure 2.**
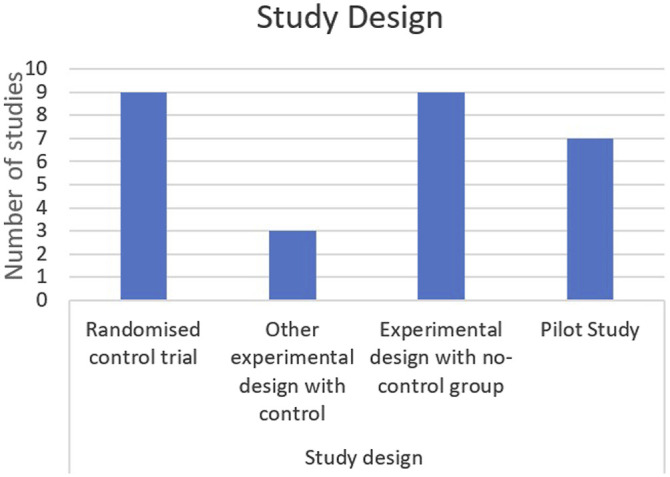
Study design

**Figure 3.**
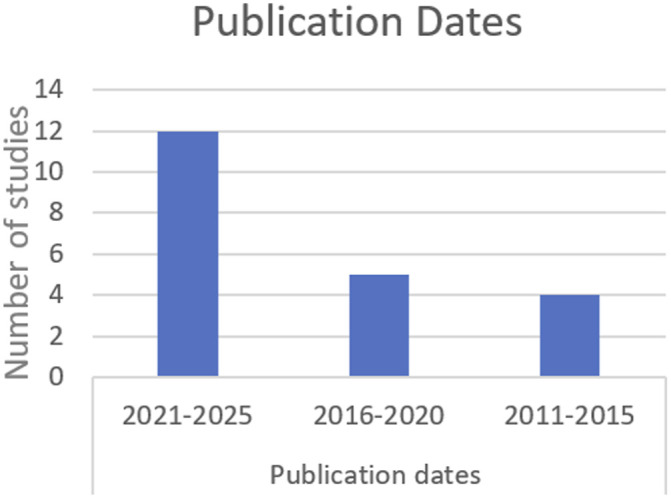
Publication dates

**Figure 4.**
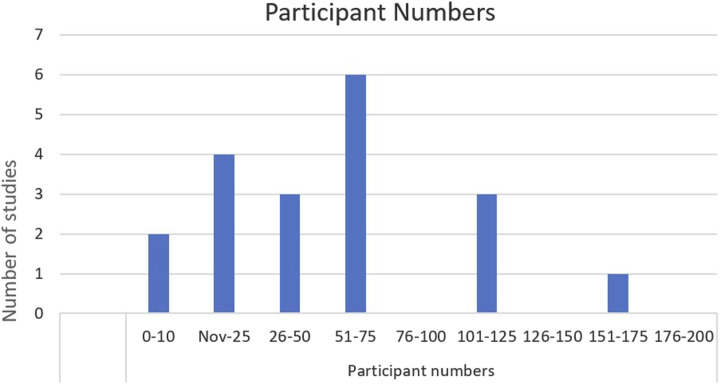
Participant numbers

**Figure 5.**
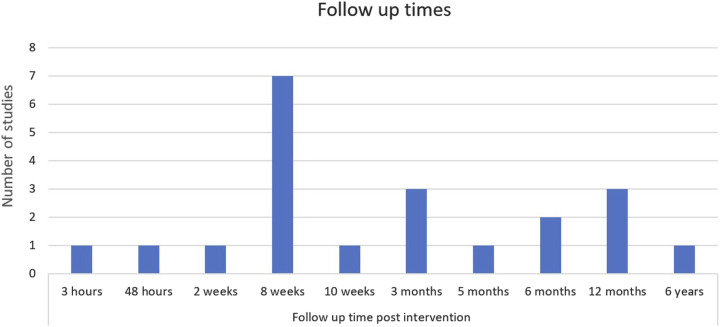
Follow-up times

**Figure 6.**
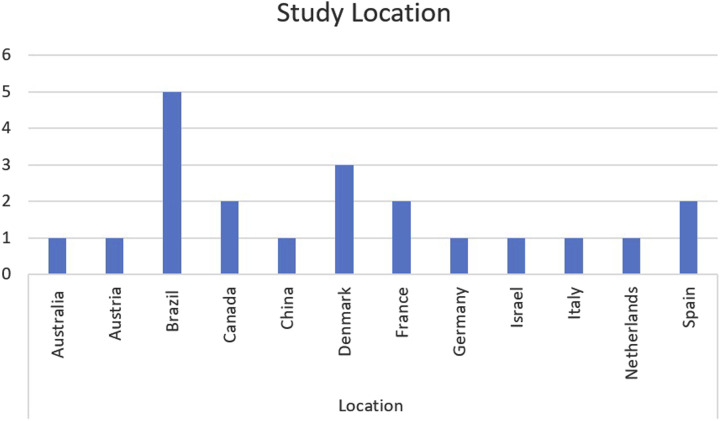
Study location

Table 2 below provides a summary of the individual characteristics of each included study. This is followed by Figures 2–6 which collate and map the characteristics of the studies found, grouped by the parameters of study design, publication date, participant numbers, follow-up time, lifestyle domain and study location. Study outcomes for each included study are then outlined in Table 3 with pain and other outcomes included.

**Table 2.** Individual Characteristics of Each Included Study.

Study, Location & design	Participants (Women with Endometriosis)	Intervention & Duration,	Comparison	Outcome Measures
Nutrition				
Cirillo et al., 2023^25^	N = 35	All participants were advised to follow a Mediterranean diet	No comparison group	**Primary outcomes:** Pain intensity (dyspareunia, non-menstrual pelvic pain, dysuria and dyschezia) utilising a VAS
Italy	*Diagnosis of endometriosis surgically or radiologically*	Follow-up after 6 months	Participants were able to continue on hormonal therapy	**Secondary outcomes:** Vitamin profiles and oxidative stress markers (such as lipid peroxidation)
Prospective study				
Marziali et al., 2012^26^	N = 207 (analysed), median age 28	Gluten free diet	No comparison group	**Primary outcomes:** Dysmenorrhoea, non-menstrual pelvic pain & dyspareunia (0 – 10 VAS)
Italy	330 recruited initially, only 207 who showed improvement in symptoms after 2 weeks completed the study	12-month follow-up		**Secondary outcomes:** Life and health satisfaction (improved physical functioning, general health perception, vitality, social functioning, mental health)
Retrospective observation case series	*Confirmed diagnosis of endometriosis radiologically and surgically*			
Ott et al., 2012^27^	N = 68	All participants were advised to follow a Mediterranean style diet for 5 months.	No comparison group	**Primary outcomes:** Change in pain (dysmenorrhoea, dyschezia, dyspareunia and dysuria) measured via an NRS.
Austria	*Surgical diagnosis of endometriosis*	Follow-up after 5 months	Participants could take non-steroids anti-inflammatory drugs. They could not take hormonal treatments.	
Prospective, experimental observational study				
van Haaps et al., 2023^28^	Low FODMAP diet (n = 22, mean age 36.9)	Low FODMAP diet or endometriosis diet (advised to avoid red meat, gluten, cow milk, sugars, nutrients high in oestrogen and limit caffeine)	No dietary change	**Primary outcomes:** Dysmenorrhoea, deep dyspareunia, chronic pelvic pain, dysuria, bloating, tiredness (0-10 VAS)
Netherlands	Endometriosis diet (n = 21, mean age 39.1)	6-month intervention duration and follow-up		**Secondary outcomes:** GI QoL questionnaire, EHP-30
Prospective study with control	Control (n = 19, mean age 37.6)			
	*Confirmed diagnosis of endometriosis either surgically or radiologically*			
Physical activity				
Artacho-Cordon et al., 2023^29^	Intervention group (n = 16, mean age 36.31)	Participants were randomly allocated into either the multi-modal tailored 9-week supervised exercise program or waitlist control.	Usual treatment from gynaecologist & offered general advice on importance of exercise	**Primary outcomes:** Quality of life (EHP – 30)
Spain	Control group (n = 15, mean age 38.40)	Program consisted of aerobic, resistance, stretching & motor control exercises adapted to each individual.		**Secondary outcomes:** Pain intensity (NRS), pressure pain thresholds, pain-related catastrophic thoughts (NRS), abdominal & back strength, lumbopelvic stability and muscle architecture
Randomised controlled trial	*Clinical diagnosis of endometriosis*	Follow-up post intervention and 1 year		
Lutfi et al., 2023^30^	3x groups: VR delivered exercises (n = 8, mean age 27)	Participants were randomised into a single 1 hr session of individualised ‘supervised’ telehealth-delivered exercise or a 1hr ‘self-managed’ virtual reality (VR) exercise session or control group	No exercise intervention	**Primary outcomes:** Acute pelvic pain – evaluated using a 100 mm VAS
Australia	Telehealth-delivered exercise (n = 8, mean age 29)	Follow-up 48 hours following intervention		
Randomised controlled trial, pilot	Control (n = 6, mean age 25)			
	*Confirmed diagnosis of endometriosis*			
Mind-body				
Goncalves, Burros, et al., 2016^31^	Treatment group (Hatha yoga) (n = 28, mean age 34.5)	Participants in the intervention group undertook 2-hour yoga sessions twice weekly for 8 weeks.	No yoga program	**Primary outcomes:** Quality of life (EHP-30) and menstrual and daily pain scale (on a VAS)
Brazil	Control group (n = 12, mean age 35.75)	Follow-up after 8 weeks		
Randomised controlled trial	*Confirmed diagnosis of endometriosis*			
Goncalves, Makuch et al., 2016^32^	N = 15, mean age 35.5	As above	No comparison group	Women’s experience of the practice of yoga and their physical and emotional state prior and post intervention.
Brazil	(15 women who had completed the 8-week yoga program participated in this qualitative study.)			Pain management.
Qualitative study conducted simultaneously with a randomised controlled trial (Goncalves et al. 2017)	*Confirmed diagnosis of endometriosis*			The role of the yoga group as psychosocial support.
Hansen et al., 2017^33^	N = 10	As below in Kold et al., 2012	N/A	**Primary outcomes:** General health status and quality of life EHP-30 and SF-36.
Denmark	*Confirmed diagnosis of endometriosis*	Follow-up 6 years after initial intervention		**Secondary outcomes:** Ongoing use of the mindfulness based interventions, overall experience of QoL and general pain in a 5-point likert scale
Follow-up of prospective observational pilot study (Kold et al, 2012^34^)				
Hansen et al., 2023^35^	MyEndo (n = 19, mean age 28.95)	MyEndo combined MBSR and ACT techniques. Delivered by a 3-hour weekly group session which included education, group therapy and a variety of mindfulness and yoga exercises.	No psychological intervention	**Primary outcome:** Pelvic pain intensity/unpleasantness measured by a 0-10 point NRS.
Denmark	Non-specific psychological intervention (n = 19, mean age 33.84)	The non-specific psychological intervention weekly group sessions involved relaxation while listening to soft, relaxation music and guided physical training.	All participants were able to continue on medical treatment as needed	**Secondary outcomes:** Endometriosis related QoL, workability, pain acceptance, and endometriosis-related symptoms.
Three armed parallel, randomised controlled trial	Wait list control (n = 16, mean age 32.81)	Follow-up after 12 weeks		
	*Diagnosis of endometriosis either surgically or* via *MRI*			
Kold et al., 2012^34^	N = 10	All participants undertook 10 weekly mindfulness-based intervention sessions (5 individual and 5 group)	No comparison group	**Primary outcomes:** General health status (SF-36) and quality of life EHP-30.
Denmark	*Confirmed diagnosis of endometriosis*	Follow-up after 12 months		
Prospective observational study, pilot				
Miazga et al., 2024^36^	N = 15, mean age 32.53	A virtual mindfulness-based stress reduction program over 8 weeks led by a social worker, incorporating core components of meditation, education on mindfulness practices and emotional regulation training along with specific endometriosis education and information regarding intimate relationships and fertility.	No comparison group	**Primary outcomes:** Pain (dysmenorrhoea, dyspareunia and chronic pelvic pain) on a VAS
Multiple-method, before and after study design	*Diagnosis of endometriosis either clinically, radiologically or surgically*	Participants were also given home exercises to practice between sessions.		**Secondary outcomes:** Quality of life (EHP-30) and pain medication use
		Follow-up after the 8 week program		A focus group was also help at the completion of the program.
Moreira et al., 2021^37^	Brief mindfulness intervention (n = 31, mean age 37.67)	Brief mindfulness intervention (4, weekly 90min group classes with mindfulness instructor) + at home tasks and practice	No mindfulness intervention	**Primary outcomes:** Endometriosis related pain (11 item pain NRS)
Brazil	Control (n = 32, mean age 34.68)	8-week intervention duration and follow-up	All participants could continue standard medical treatment	**Secondary outcomes:** QoL (SF-36) and stress perception (PSS)
Randomised controlled trial, pilot	*Diagnosis of deep endometriosis* via *MRI*			
Ravins et al., 2023^38^	N = 42, mean age 30.42	All participants completed 8 weeks of conventional therapy followed by 8 weeks of twice weekly 90-minute endometriosis yoga classes.	No comparison group	**Primary outcomes:** Stress & QoL (EHP-30)
Israel	*Confirmed diagnosis of endometriosis*	Follow-up after the yoga intervention – 2 months		**Secondary outcomes:** Pain and intensity of bleeding during last menstrual period.
AB (baseline then intervention) study design, pilot				
Other or multi-modal				
Bi & Xie, 2018^39^	Neuromuscular electrical stimulation (NMES) (n = 83, mean age 31.6)	The treatment group undertook NMES therapy for 30mins, 3x weekly for 10 weeks.	No NMES	**Primary outcomes:** Pain measured by NRS and ESSS – which required participants to rate dysmenorrhoea, dyspareunia and non-mensural pain.
China	Wait list control (n = 71, mean age 32.2)	The gel pads for the NMES were applied with bilateral acupoints of Sanyinjiao (above the medial malleolus), Zhongji (medial lower abdomen, 4 cm below umbilicus) and guanyuan (3 cm below umbilicus)		**Secondary outcomes:** Quality of life utilising the SF-36
Retrospective study	*Histological confirmation of endometriosis*	Follow-up after 10 weeks		
Breton et al., 2025^40^	The School of Endo (n = 146, mean age 36.7)	The digital tool (*The School of Endo*) was developed using a cognitive behavioural therapy approach (CBT) based on the endometriosis health profile (EHP) items. It focused on 5 non-pharmacological interventions of disease education (including pain mechanism), diet, adapted physical activity, well-being and mental-health and sexual health. There was a range of content including videos, exercises, written content, live sessions, quizzes and a community-based platform.	No access to *The School of Endo*	**Primary outcomes:** A NRS from 1-11 was used for the level of overall pain, anxiety, depression, dysmenorrhoea, dyspareunia, dyschezia, dysuria, chronic pelvic pain, gastro-intestinal disorders, chronic fatigue, neuropathic pain, and endo belly.
France	Control group (n = 149, mean age 36.6)	3 month follow-up		**Secondary outcomes:** The EHP-5 was also recorded.
Cohort study, pilot	*Diagnosis of endometriosis clinically, surgically or radiologically*.			
De Hoyos et al., 2023^41^	Environmental enrichment (EE) intervention (n = 29, mean age 32.7)	The EE intervention comprised six fortnightly modules which involved the intervention group undertaking a support group meeting, followed by a novel stress-management activity in an open space. Activities included yoga, yogic breathing, mindfulness, aromatherapy, art therapy, drama therapy and dance therapy while locations included the beach, lake, garden, hot springs and countryside.	No environmental enrichment	**Primary outcomes:** Pelvic pain (dysmenorrhoea, dyspareunia, chronic pelvic pain) on a 1-10 NRS
Spain	Waitlist control (n = 27, mean age 34)	3 month follow-up	Both groups could continue on standard analgesic, hormonal, surgical or psychological therapy as needed.	**Secondary outcome:** Quality of life (EHP-30), stress (PSS), anxiety (GAD-7), depression (PHQ) and pain catastrophising (PCS)
Randomised controlled trial, pilot	*Diagnosis of endometriosis surgically*			
Merlot et al., 2023^42^	Virtual reality headset (n = 51, 20 of which had adenomyosis; mean age 33.7)	Virtual reality Endocare program (visual and auditory components) used twice daily on the 5x consecutive most painful days of the month	Sham program	**Primary outcomes:** Pain perception on a 1-10 NRS
France	Control (n = 51, 16 of whom had adenomyosis; mean age 32.1)	Follow-up – 60mins, 120mins & 180mins post intervention	All participants were able to use pain medication if needed	**Secondary outcomes:** General pain, stress, fatigue, medication intake, QoL (EHP-5)
Randomised controlled trial	*Confirmed diagnosis of endometriosis or adenomyosis*			
Mira et al., 2015^43^	Group 1 was ‘acupuncture-like’ TENS (frequency 8 Hz, pulse duration 250us) – (n = 11, mean age 41.0)	The TENS was applied in the S3/4 region in both groups.	Acupuncture-like TENS	**Primary outcomes:** Pain (chronic pelvic pain, dyschezia, dysuria and dysmenorrhoea) utilising a VAS. Deep dyspareunia using the DDS
Brazil	Group 2 self-applied TENS (frequency 85 Hz, pulse duration 75us) – (n = 11, mean age 30.9)	Acupuncture-like TENS occurred in 30min, weekly sessions for 8 weeks.	All participants could continue on hormonal therapy.	**Secondary outcomes:** QoL (EHP-30)
Randomised controlled trial	*Diagnosis of deep endometriosis* via *ultrasonography*	Self-applied TENS occurred for 20mins twice daily for 8 weeks.		
		Follow-up after 8 weeks		
Mira et al., 2020^44^	Intervention (hormonal therapy + electrotherapy) (n = 53, mean age 35.06)	TENS therapy applied to the parasacral region for 20mins twice daily for 8 weeks.	No TENS therapy	**Primary outcomes:** Chronic pelvic pain utilising a VAS (for chronic pelvic pain, dyschezia, dysmenorrhoea) and deep dyspareunia (via DDS).
Brazil	Control (standard hormonal therapy) (n = 48, mean age 37.21)	Follow-up after 8 weeks of therapy	All participants could continue on hormonal therapy.	**Secondary outcomes:** QoL (EHP-30) and sexual function (FSFI).
Randomised controlled trial	*Diagnosis of deep endometriosis* via *ultrasonography or MRI*			
Rohloff et al., 2024^45^	N = 106, mean age 33	Participants were advised to use the EndoApp – with features such as an endometriosis diary, exercise guides, nutrition advice, educational articles and videos, psychological support, stress-reducing concepts and guidance on positive coping.	No comparison group	**Primary outcomes:** QoL utilising the EHP-30 and quality of life-index.
Germany	(n = 64 reported using the app and n = 42 didn’t use the app in the 2 week period)	Follow-up after 2 weeks		
Observational, pilot study	*Confirmed diagnosis of endometriosis*			

Table 2 acronyms: FODMAP – fermentable oligosaccharides, disaccharides, monosaccharides and polyols, VAS – visual analogue scale, GIQoL – gastro-intestinal quality of life, EHP – endometriosis health profile, NRS – numeric rating scale, PSS – perceived stress cale, SF-36 – short form health survey, GAD – generalised anxiety disorder 7, PHQ – patient health questionnaire 8, PCS – pain catastrophising scale, PPT – pressure pain thresholds, FSFI – female sexual function index, DDS – deep dyspareunia scale, ESSS – endometriosis symptom severity score.

**Table 3.** Summary of Outcome Measures of Included Studies.

	Intervention + study type	Outcome	Reference
Nutrition	Low FODMAP diet or	**Pain outcomes:** All participants adhering to dietary intervention reported significantly less dyspareunia, dysuria, bloating and tiredness after adhering to the diet for 6 months compared to their baseline (range *P* < 0.001 to *P* = 0.012).	Van Haaps et al., 2023^28^
	Endometriosis diet vs control group	When compared to the control group, deep dyspareunia in the low FODMAP group (*P* = 0.075) and bloating in the endometriosis group (*P* = 0.041) remained statistically significant.	
	*Prospective study with control group*	**Other outcomes:** All participants adhering to diet scored significantly better on the QoL domains of pain, powerlessness, emotional well-being, self-image, work life and sexual intercourse after 6 months compared to their baseline.	
		When compared to the control group, the only statistically significant QoL result was in social support and medical profession.	
	Gluten free diet	**Pain outcomes:** At 12 months follow-up, 75% of patients reported statistically significant improvement in painful symptoms (*P* < 0.005).	Marziali et al., 2012^26^
	*Retrospective observational case series*	**Other outcomes:** There was a significant increase in score of all domains of physical functioning, general health perception, vitality, social functioning and mental health.	
	Mediterranean diet	**Pain outcomes:** The intention-to-treat analysis demonstrated a significant improvement in general pain based on NRS (4.2±2.5 pre-intervention to 2.5±2.4 post intervention; *P* = 0.003).	Ott et al., 2012^27^
	*Prospective study*	Patients also experienced significant improvement in dysmenorrhoea (*P* < 0.001), dyspareunia (*P* = 0.011) and dyschezia (*P* = 0.032).	
	Mediterranean diet	**Pain outcomes:** At 3 months, patients reported reduction in dyspareunia (*P* = 0.04), non-menstrual pelvic pain (*P* = 0.06), dysuria (*P* = 0.04), and dyschezia (*P* < 0.001). Dyspareunia (*P* = 0.002) and dyschezia (*P* < 0.001) were even more significantly reduced after 6 months of adherence to the Mediterranean diet.	Cirillo et al., 2023^25^
	*Prospective study*		
Physical activity	‘Physio-EndEA’	**Pain outcomes:** There was a statistically significant improvement in pain on the EHP-30:	Artacho-Cordon et al., 2023^29^
		- Pre-intervention – 50.30+/− 22.71 intervention vs 54.40+/−16.79 control	
		- Post-intervention – 29.53+/−16.17 intervention vs 47.72+/−19.75 control (*P* < 0.05, Cohen d > 0.8)	
		−1 year – 34+/−23.15 intervention vs 56.18 +/−15.27 control (*P* < 0.001, Cohen d > 0.8)	
		-	
	*Randomised controlled trial*	There was close-to-statistically significant improvement in current pelvic pain and dyschezia on NRS between intervention and control post intervention. A statistically significant improvement was found in dyspareunia post intervention; however, this was not maintained at 12 months.	
		**Other outcomes:** When analysing the EHP-30, there was a statistically significant improvement in total score, pain and emotional well-being which persisted at the 1 year follow-up (all large effect with Cohen d > 0.8).	
		There was a significant improvement on the pain catastrophizing scale post intervention and at 12 months.	
		Pressure pain thresholds improved in both pelvic and distal nociceptive sites in the intervention group. Improvements in trunk strength and lumbar-pelvic stability were also observed.	
	Single episode telehealth-delivered exercise intervention or unsupervised VR exercise intervention	**Pain outcomes:** There was no significant pain VAS score change between the groups following the training intervention.	Lutfi et al., 2023^30^
		There was an increase in pelvic pain scored in baseline across the three groups, however both intervention groups showed a lower magnitude increase in pain score:	
		• VR increase +9 +/−24 mm	
	*Randomised controlled trial* – *pilot*	• Telehealth +10 +/−12 mm	
		• Control +16 +/− 12 mm.	
Sleep			
Mind-body	Brief mindfulness intervention	**Pain outcome:** bMBI statistically significantly improved the primary outcomes of pelvic pain (*P* = 0.013, Cohen’s f^2^ = 0.161), dyschezia (*P* = 0.007, Cohen’s f^2^ = 0.232) and pain unpleasantness (*P* = 0.000, Cohen’s f^2^ = 0.676) post treatment in the intervention group compared to control.	Moreira et al., 2021^37^
	*Randomised controlled trail – pilot*	**Other outcomes:** bMBI also improved the SF-36 vitality (NNT 2.1, Cohen’s f^2^ = 0.22) and SF-36 mental health (NNT 3.5 and Cohen’s f^2^ = 0.34) compared to control. There was only marginal non-significant effect of the bMBI on stress perception reduction.	
	Endometriosis yoga (twice weekly online yoga classes for 8 weeks)	**Pain outcome:** Pelvic pain improved by 0.97 +/− 0.32 on a numeric pain rating scale (*P* = 0.01). The pain component of the EHP-30 changed by 12.33 +/− 2.80 (*P* = 0.001) on the 100-point scale post intervention.	Ravins et al., 2023^38^
	*AB study design* – *Pilot study*	**Other outcomes:** The EHP-30 score improved by 10.43 +/− 2.59 (*P* = 0.001). The variables of pain, control and powerlessness, emotional well-being, social support, work and intercourse improved the most.	
	Hatha Yoga	**Pain outcome:** At the end of the 8-week program, the EHP-30 pain domain improved from a mean of 60.80 to 32.39 in the intervention group compared to a change of 58.71 to 55.05 in the control group, showing statistical significance (*P* = 0.0046).	Goncalves, Burros, et al., 2016^31^
	*Randomised controlled trial*	The pain VAS score showed a statistically significant improvement in the intervention group (*P* = 0.0007).	
		**Other outcomes:** The quality-of-life domains of importance, well-being and image also improved significantly (*P* = 0.0006, *P* = 0.0009 and *P* = 0.0087 respectively)	
	Hatha Yoga	**Pain:** All participants reported that yoga was beneficial to control pelvic pain.	Goncalves, Makuch, et al., 2016^32^
	*Qualitative study conducted simultaneously with a randomised controlled trial*	**Other outcomes:** Women identified a relationship between pain management and breathing techniques.	
	MyEndo	**Pain outcome:** Compared to control group, the psychological intervention (MY-ENDO or non-specific) did not demonstrate a statistically significant change in pelvic pain intensity or pelvic pain unpleasantness (once adjustments were made for medication use).	Hansen et al., 2023^35^
	Non-specific psychological intervention	**Other outcomes:** Statistically significant changes were seen in the QoL domains of ‘control and powerlessness’ (*P* = 0.019), ‘emotional wellbeing’ (*P* = 0.003) and ‘social support’ (*P* = 0.042).	
	*Three armed parallel, multi-centre randomised controlled trial*	There was no statistically significant difference between the two intervention groups.	
	Mindfulness-based intervention	**Pain outcome:** The EHP-30 domain of pain improved significantly from 52.53 pre-intervention to 33.18 post intervention, 31.59 at 6mths and 28.12 at 12 months (*P* = 0.003).	Kold et al., 2012^34^
		**Other outcomes:** All domains of the EHP-30 improved with the intervention and remained improved at 6- and 12-month follow-up (except for self-image where improvement was temporary).	
	*Prospective observational study* – *Pilot study*	**Pain outcome:** 8 out of 10 patients experienced a better or much improved pain level compared to prior the intervention.	Hansen et al., 2017^33^
		**Other outcomes:** When comparing data from the 12 months follow-up with data from the 6-year follow-up, results showed no significant differences in mean scores on all scales of the EHP-30 and almost all scales of the SF-36 scale scores (and thus improvements seen at 12 months were sustained at 6 years follow-up)	
	Virtual mindfulness-based stress reduction program	**Pain outcome:** There was no significant change in pain scores (dysmenorrhoea, dyspareunia or other times) or medication use following the intervention.	Miazga et al., 2024^36^
	*Multiple-method, before & after study design*	**Other outcomes:** There was a statistically significant increase in control and powerlessness (*P* = 0.012), emotional well-being (*P* = 0.048), social support (*P* = 0.030) and self-image (*P* = 0.014) following the MBSR program on the EHP-30.	
Social connection	Virtual mindfulness-based stress reduction program	Participants found the sense of community, education and application of mindfulness tools when applied to pain to be the most beneficial components of the intervention. They found therapeutic benefits from sharing their experiences with the disease.	Miazga et al., 2024^36^
	Hatha Yoga	Most of the participants reported benefits with forming social connections with other women in the yoga program. They reported that this allowed them to reinterpret their beliefs regarding pain associated with endometriosis and recognise that their pain was real and develop strategies for managing their pain.	Goncalves, Makuch, et al., 2016^32^
	*Qualitative study conducted simultaneously with a randomised controlled trial*		
Substance avoidance			
Other	Environmental enrichment (web-based module program)	**Pain outcome:** The intervention and control groups showed similar and not statistically significant change in global pain impact scores at baseline and end of study.	De Hoyos et al., 2023^41^
	*Randomised controlled trial* – *Pilot study.*	**Other outcomes:** There was a statistically significant improvement in GAD-7 in the intervention group at the end of the intervention (*P* = 0.006) and 3 months after (*P* < 0.0001). Similarly, there was improvement in depressive symptoms in the PHQ-8 with lower levels in the intervention group (*P* = 0.014 end of intervention, *P* = 0.006 3 months post).	
	Virtual reality ‘Endo-care’ program used during days of maximum pain in menstrual cycle	**Pain outcome:** Pain intensity reduction was significantly higher in the Endocare Group on Day (D)1, D2 and D3 compared to control. Pain intensity reduction reached a maximum on D2 with a 51.6% reduction at 120mins post intervention and 51.2% reduction at 180mins in the Endocare group vs 21.2% and 23.9% respectively in the control group. There was no difference in pain intensity reduction observed in the groups on D4 and D5.	Merlot et al., 2023^42^
		There was no significant difference in pain between intervention and control at wakeup and bedtime.	
	*Randomised controlled trial*	**Other outcomes:** There was no significant difference in fatigue and stress or quality of life between the two groups (both had reduction in fatigue and stress from D1 to D5).	
	*The School of Endo* digital tool (diet, disease education, physical activity, well-being, mental health and sexual health + community-based platform)	**Pain outcome:** Actively following the digital program for 3 months was associated with an improvement in neuropathic pain (improving in 41% vs 23.1%, *P* = 0.02), and endo belly perception (improving in 41% vs 24.5%, *P* = 0.03) among program participants when compared to the control group. There was no significant change in dysmenorrhoea, dyspareunia, dyschezia, dysuria or chronic pain.	Breton et al., 2025^40^
		**Other outcomes:** Actively following the digital program for 3 months was associated with a significant improvement in global symptom burden (improving in 20% vs 6.1%, *P* = 0.003), anxiety (improving in 42% vs 26.5%, *P* = 0.002) & depression (deteriorating in 10% vs 27.9%, *P* = 0.003) among program participants when compared to the control group.	
	*Cohort study – Pilot study*	Active program participants also showed an improvement in their QoL at 3 months and significant improvement in knowledge on endometriosis.	
	‘Endo-app’	**Pain outcome:** There was a statistically significant improvement in the EHP-30 domain of pain with improved by 9.23 points (Cohen’s d = 0.73) in those who used the Endo-App.	Rohloff et al., 2024^45^
	*Observational study – Pilot study*	**Other outcomes:** There was a statistically significant improvement in quality of life following two weeks of use of the Endo-App. The EHP-30 domains of work-life, control and helplessness and pain improved the most, while self-image only improved a small amount and was not statistically significant.	
	Self-applied TENS (20mins 2x daily for 8 weeks)	**Pain outcome:** There was a statistically significant change in chronic pelvic pain and deep dyspareunia between the intervention and control groups following the 8 weeks TENS intervention:	Mira et al., 2020^44^
		- Chronic pelvic pain VAS decrease 36% in the intervention group compared with 3.68% in the control	
		- Deep dyspareunia decreased 32.67% in the intervention group compared with 13.84% in the control	
	*Randomised controlled trial*	The number of days each week of pelvic pain also improved from 3.27 to 2.22 (*P* = 0.028, 32.11% decrease) in the intervention group.	
		**Other outcomes:** There was an improvement in QoL across all domains of the EHP-30 in the intervention group and suggested improvement in sexual function.	
	TENS – acupuncture-like TENS vs self-applied	**Pain outcome:** For women in both intervention arms, TENS provided symptomatic pain relief, with significant differences before and after chronic pelvic pain treatment (*P* < .0001), deep dyspareunia (*P* = 0.001) and dyschezia (*P* = 0.001).	Mira et al., 2015^43^
		There was no significant improvement in dysmenorrhoea or dysuria.	
	*Randomised controlled trial*	**Other outcomes:** The EHP-30 demonstrated improvement in pain, control & powerlessness, emotional well-being, social support and self-image domains.	
		There was no significant difference between the two treatment arms.	
	Neuromuscular electrical stimulation (NMES)	**Pain outcome:** At 5 weeks, there was no significant difference in pain measured by the 10-point NRS in those that had received NMES compared with control:	Bi & Xie, 2018^39^
		- Intervention group pain dropped by −1.4 (−2.0, −0.7) compared with control−0.5 (−0.7, −0.3); *P* = 0.14.	
		After 10 weeks of intervention there was statistically significant improvement in all outcome measures in the intervention group compared with control. Pain on the NRS:	
	*Retrospective study with control*	- Intervention group pain dropped by −2.9 (−3.7, −1.8) compared with control −0.6 (−1.0, −0.3); *P* = 0.02.	
		**Other outcomes:** At 5 weeks, there was no significant difference in ESSS or quality of life measured by the SF-36.	
		After 10 weeks there was statistically significant improvement in all outcome measures in the intervention group compared with control.	

Table 3 acronyms: FODMAP – fermentable oligosaccharides, disaccharides, monosaccharides and polyols, VAS – visual analogue scale, GIQoL – gastro-intestinal quality of life, EHP – endometriosis health profile, NRS – numeric rating scale, PSS – perceived stress cale, SF-36 – short form health survey, GAD – generalised anxiety disorder 7, PHQ – patient health questionnaire 8, PCS – pain catastrophising scale, PPT – pressure pain thresholds, FSFI – female sexual function index, DDS – deep dyspareunia scale, ESSS – endometriosis symptom severity score.

Table 3 below provides a summary of the outcomes of each included study. This includes pain outcomes and other domains (such as quality of life).

All 21 studies included consisted of different methodologies. Nine were randomised controlled trials, three were other experimental design with a control group while the remaining nine did not have a control comparison. Seven were pilot studies. Participants in each study were women over the age of 18 with a diagnosis of endometriosis. This diagnosis was either clinical, or through imaging (ultrasound or MRI) or surgery. One study included participants with a diagnosis of adenomyosis.^42^ Participant numbers varied from ten to 300, with 50% of studies including less than 30 participants. The follow-up time ranged from 3-hours to 6 years post intervention. There were four studies analysing the impact of the lifestyle domain of nutrition, two for physical activity, eight for mind-body interventions and seven for other interventions. Two of the articles analysed the impact of social connection as a secondary outcome.

Positive results obtained with the following interventions demonstrated a statistically significant positive impact on pelvic pain management in women with endometriosis:

- Nutrition – The low FODMAP diet and dyspareunia,^28^ Mediterranean diet and dyspareunia, non-menstrual pelvic pain, dysuria and dyschezia,^25,27^ gluten free diet and pain symptoms^26^
- Physical activity – ‘Physio-EndEA’ and dyspareunia^29^
- Mind-body – endometriosis yoga on pain.^32,38^.
- Other interventions – The virtual reality program ‘Endocare’ on acute pain intensity,^42^ ‘School of Endo’ digital health tool on neuropathic pain,^40^ ‘Endo-App’ on pain,^45^ TENS on pain,^39^ chronic pelvic pain, dyspareunia and dyschezia^43,44^

## Discussion

This scoping review returned 21 relevant studies which examined the impact of different lifestyle interventions in pain management in women with endometriosis and/or adenomyosis. The 21 studies all varied in terms of methodology, participant numbers, intervention and follow-up time. This heterogenicity between studies impacts the ability to develop comparisons between interventions while the methodologies and low participant number impact the external validity of the results obtained.

Despite this, the positive pain outcomes identified are consistent with other chronic pain research which has demonstrated positive impact with nutritional,^46^ physical activity^47^ and mind-body^31,38^ interventions, however, further well-designed, rigorous, and large-scale trials are also required in this chronic pain area.

There were mixed results from mindfulness-based interventions. With two studies demonstrating a positive impact from mindfulness-based intervention on pain (Kold et al. (2012) and Moreira et al. (2022))^34,37^ while two studies (Hansen et al. (2023) and Miazga et al. (2024)) demonstrated no improvement on pain.^35,36^ Miazga et al. (2024) hypothesises that the limited impact on pain in their study may be due to participants experiencing chronic pain for an average of 13 years and thus the neurological changes that occur with chronic pain may require a longer intervention with mindfulness to build new neuronal pathways.^36^ The participants in Hansen et al. (2023) also had an average length of pain of 13 years prior to the intervention while those in the study by Moreira et al. (2022) only had a 7-year history of pain.^35,37^ There was no chronic pain timeframe recorded in the study by Kold et al. (2012).^34^

Given the low risk associated with many of the included intervention, clinicians may consider discussing the outcomes with their patients with endometriosis and including them as part of an individualised patient-centred management plan. A proposed management plan is included in the Table 4 below. Further research is required before this is included in population-wide guidelines.

**Table 4.** Suggested Management Plan Clinician May Choose to Implement With Their Patients.

Intervention	Specific Intervention	Potential Benefit	Resources
Mediterranean diet	Adherence to the Mediterranean diet	Reduces bloating, pelvic pain, tiredness; may improve emotional well-being	Dietician
			Mediterranean diet handouts
Low FODMAP diet (particularly for women with bowel symptoms)	8-weeks low-FODMAP diet, then work with dietician to re-introduce and monitor symptoms	Reduces bloating, pelvic pain, tiredness; may improve emotional well-being	Dietician
Physical activity	8-week Personalised strength & aerobic exercise program	Improves pain, trunk strength, stability, emotional well-being	Exercise physiologist
			Physiotherapist
Mindfulness	8-week mindfulness based intervention education program	Improves pain, trunk strength, stability, emotional well-being	Psychologist with special interest in mindfulness-based therapy
	Ongoing individual mindfulness based practice at home – 10-20 mins daily		Online mindfulness based apps
Yoga	8-week, twice weekly yoga program	Decreases pelvic pain, improves QoL	In-person yoga studio
	Ongoing yoga practice at home 2x weekly		Online yoga videos
TENS	Self-applied TENS to the parasacral region for 20mins twice daily for 8 weeks (frequency 85 Hz, pulse duration 75us)	Decreases pelvic pain, improves QoL	TENS machine
	Ongoing use in an as-needed basis		
Multi-modal	Utilisation of the ‘Endo-App’ for education and monitoring of symptoms	Enhances symptom awareness, improves QoL, and reduces anxiety	Utilisation of the ‘Endo App’ https://endometriose.app/en/endo-app/

### Research Gaps & Future Priorities

Significant research gaps have been identified. Firstly, there were no interventional trials identified examining sleep, or substance cessation interventions on pain associated with endometriosis. This is despite many observational studies illustrating that often women with endometriosis experience poor quality sleep and high levels of insomnia.^48,49^ It is also known that poor sleep has a direct impact on inflammation and the pain response.^50^ Consequently, it can be hypothesised that interventions which aim to improve sleep quality and quantity in women with endometriosis may be associated with improvement in pain.

Similarly, there have been studies which illustrate an association between moderate alcohol use and endometriosis development.^51^ Alcohol is also known to be associated with increased inflammation.^52,53^ Thus, it could also be hypothesised that decreasing alcohol intake may improve pain associated with the chronic inflammatory condition of endometriosis.

Adenomyosis was only included as a diagnosis in one of the included studies despite at times co-existing with endometriosis and impacting between 8.8% and 61.5% of women.^6^ Given that adenomyosis and endometriosis share similar features, it is predicted that many of the lifestyle interventions which show positive outcomes in endometriosis may show similar results in adenomyosis. However, intervention trials involving patients with only adenomyosis would be paramount to confirm this hypothesis. This is particularly important when considering non-pain outcomes (such as infertility), given current fertility sparing treatment for adenomyosis is largely limited to hormonal options.

Both endometriosis and adenomyosis are chronic conditions impacting women of childbearing age with symptoms potentially lasting from menarche to menopause. Despite this, most of the intervention trials included had a follow-up period of less than 6 months. It should be a priority for future research to have longer follow-up periods or follow-up studies conducted to analyse the long-term impact of specific lifestyle interventions on these chronic conditions.

It is interesting to consider the developing role of digital media in the lifestyle management of endometriosis and/or adenomyosis. There were five studies identified which utilised digital programs as intervention, and all were published since 2023.^30,36,40,42,45^ A digital program has the possibility of impacting a greater number of endometriosis sufferers and does not require any initial or ongoing allied heath support which also improves access.

Digital programs also have the possibility of being multi-modal, covering different lifestyle pillars with education and interventions across the breadth of lifestyle medicine. Given that all lifestyle pillars included in this review demonstrated some positive outcomes, it can be hypothesised that multi-modal lifestyle interventions may be effective in long-term management in endometriosis and adenomyosis. This was demonstrated by Breton et al. (2025)^40^ and Rohloff et al. (2024)^45^ both of which had a multi-modal intervention and reported positive impacts on both pain and quality of life. This multi-modal lifestyle approach is consistent with current recommendations for the management of chronic pain.^54^ Further well-designed, rigorous, and large-scale randomised controlled trials looking at multi-modal interventions in the pain management of endometriosis and adenomyosis are vital.

Of the intervention trials included in this review, seven were pilot studies, six of which were published within the previous three years. This is over one quarter of the studies included. This may demonstrate that there is growing interest in the lifestyle management of endometriosis and thus in subsequent years there may be further, higher quality, intervention trails published in this area. Consistency in methodology and intervention in these future studies would allow for the completion of systematic reviews and meta-analysis.

### Strengths & Limitations

There are several limitations to this review. Firstly, as outlined in the introduction, endometriosis can cause not only pelvic pain, but also negatively impact other factors such as fertility, quality of life, mental health or need for repeat surgery. A limitation of this scoping review is the focus on pain management as the primary outcome. Many of the studies analysed the secondary outcomes of quality of life and mental health and illustrated significant benefits, however, these outcomes were not the focus of this review. Given the search protocol and inclusion criteria were limited to studies with pain as an outcome, studies with only non-pain outcomes were either not identified during the search or excluded during the screening process. Consequently, there may be other published interventional research which demonstrates benefits in outcomes, such as quality of life, fertility or mental health, in women with endometriosis which were not included in this review. This is clinically relevant as improvement in these outcomes are likely to be of interest to endometriosis or adenomyosis sufferers. Of the studies included in this review, there were three studies included which demonstrated improvement in quality of life or mental health domains despite no impact on pelvic pain including the environmental enrichment program^41^ and two psychological interventions.^35,36^ When developing future review or intervention trials it would also be important to expand the analysed outcomes of lifestyle interventions to include factors such as fertility, surgery use, quality of life and mental health in addition to the pain symptoms.

Secondly, the database search for this scoping review was limited to only English language studies. This is a limitation as it does not include the whole scope of the topic as there may have been non-English trials conducted and published. Given the breadth of lifestyle medicine, developing a search protocol which covered all the pillars and self-management strategies may be challenging. The authors feel that the included search was comprehensive however it is possible that an alternative search protocol may harness different results. Two additional papers were identified and incorporated when hand-searching the reference list of originally included studies from the search protocol.

Finally, title, abstract and full text screening was conducted by two authors (BH & ND) however data extraction was only conducted by one author (BH). Previous studies have demonstrated a 10.76% error rate by human reviewers during abstract screening.^55^ Having two reviewers decreases this error rate.^56^ Consequently, having two authors involved in abstract and full text screening is a strength of this study as it reduces the risk of errors during study selection. However, having only one author for data extraction is a limitation as studies have illustrated a high rate of data extraction errors of up to 50%.^57^ Thus, it is recommended to have dual-reviewer data extraction to reduce this risk of error.^58^ There is a risk of bias in this review with the potential for biased results and wrong conclusions drawn.

## Conclusion

This report aimed to retrieve and examine the evidence for lifestyle interventions in the management of pelvic pain associated with endometriosis and/or adenomyosis. 21 relevant articles were retrieved which varied significantly in trial design, participant numbers and follow-up. Outcomes illustrated potential effectiveness for dietary changes, physical activity, brief mindfulness interventions, yoga, multi-modal digital programs and TENS, however due to study heterogeneity and often low participant numbers, definitive conclusions cannot be drawn.

Further research is paramount in this area and should also be extended to include further outcome domains in addition to pain, such as quality of life, fertility and mental health. It will be equally important to include interventions involving the lifestyle pillars of sleep and substance use. Participants with adenomyosis must also be included in future research.

Future well-designed, rigorous, and large-scale randomised controlled trials studies would allow for systematic reviews or meta-analysis to be undertaken and ensure high quality evidence is collated. This could lead to updated guidelines, and improve care, for the many women suffering from endometriosis or adenomyosis.

## Supplemental Material

Supplemental Material - What is the Evidence on Lifestyle Interventions for the Symptom Management of Pelvic Pain in Women With Endometriosis or Adenomyosis? A Scoping Review Supplemental Material for What is the Evidence on Lifestyle Interventions for the Symptom Management of Pelvic Pain in Women With Endometriosis or Adenomyosis? A Scoping Review by Bethany Hough, Sam Manger, Natalie Drever in American Journal of Lifestyle Medicine

## Data Availability

The data that support the findings of this study are available from the corresponding author, BH, upon reasonable request.*
