# Supplementary material for: What is the Evidence on Lifestyle Interventions for the Symptom Management of Pelvic Pain in Women With Endometriosis or Adenomyosis? A Scoping Review
Source: Am J Lifestyle Med. 2026 Feb 24:15598276261419770. Online ahead of print. doi: 10.1177/15598276261419770 (PMC12935590; doi:10.1177/15598276261419770)
Supplement: Supplemental Material - What is the Evidence on Lifestyle Interventions for the Symptom Management of Pelvic Pain in Women With Endometriosis or Adenomyosis? A Scoping Review [file sj-pdf-1-ajl-10.1177_15598276261419770.pdf]

Appendix A – Table of proposed search strategy

	MeSH terms – OVID	MeSH terms - CINAHL	Key word terms - Scopus
**Condition terms (combined with OR)**	Endometriosis<br>Adenomyosis	Endometriosis<br>Adenomyosis	Endometrio*<br>Adenomyo*
**Pillar Terms (all combined with OR)**			
Nutrition	exp 'diet, food, and nutrition'<br>exp 'nutrition therapy'<br>exp 'diet'<br>Nutritionist	Diet+<br>Nutrition<br>Nutrition services+<br>Nutritionists<br>Dietitians	Diet*<br>Nutrition*<br>Food
Physical activity	exp 'exercise'<br>exp 'exercise movement techniques'<br>exp 'exercise therapy'<br>exp 'sports'<br>dancing	Exercise+<br>Exercise intensity<br>Physical activity<br>Physical fitness<br>Sports+<br>Dancing+	Exercise*<br>Physical activity<br>Danc*<br>Sport*<br>Athletic*<br>Stretch*
Sleep	exp Sleep<br>Rest	Sleep+	Sleep*
Mind-body	exp 'mind-body therapies'<br>exp 'mindfulness'	Mind-body techniques +<br>Mindfulness+	Mind-body*<br>Breathing<br>Yoga<br>Meditation<br>Relax*<br>Nature<br>Imagery*<br>Reverie*<br>Mindfulness*
Social connection	exp 'self-help groups'<br>exp 'social group	Social group<br>Support group	Support group<br>Social*

	community networks<br>exp ‘social support’<br>social isolation<br>social media	Social isolation<br>Support, social +<br>Social media	Community*
Substance cessation	Smoking cessation<br>Smoking reduction<br>Tobacco use cessation<br>Illicit drugs<br>Exp ‘Drinking behaviour’	Substance abstinence +<br>Smoking cessation<br>Smoking cessation programs<br>Street drugs	Smok*<br>Illicit drug<br>Recreational drug<br>Street drug<br>Illegal drug<br>Alcohol*<br>Ethanol*
Other terms	Self-care<br>Relaxation<br>Work-life balance<br>Quality of life<br>Internet-based intervention<br>Self-management	Wilderness experience<br>Life-style changes<br>Self-care<br>Work-life balance<br>Internet-based intervention<br>Self-management	Self-help<br>Self-care<br>Heat pack<br>Lifestyle<br>Internet intervention<br>Self-management
	Key words OVID & CINAHL		
	Heat pack		
**Outcome terms (all combined with OR)**	Pelvic pain<br>Dysmenorrhoea<br>Dyspareunia<br>Dyschezia (keyword)<br>Exp ‘Pain perception’<br>Pain<br>Acute pain<br>Chronic pain<br>Interstitial cystitis	Pelvic pain +<br>Pain<br>Dyspareunia<br>Chronic pain<br>Dyschezia (keyword)<br>Visceral pain<br>Interstitial cystitis	Pain* AND menstru*<br>Interstitial AND cystitis<br>Acute AND pain<br>Chronic AND pain<br>Dyspareunia<br>Dyschezia<br>Pelvic AND pain<br>Pain
**Combine collated conditions ‘AND’ collated pillar terms ‘AND’ collated outcome terms**			

Appendix B – Full data extraction table

Author /year	Country	Aim/purpose	Study type/methodology	Population & sample size<br><br>Lifestyle Pillar	Intervention – type, details, duration, comparator	Outcomes	Key findings relating to scoping review question
Artacho-Cordon et al., 2023	Spain	To evaluate the effectiveness of a multi-modal 9-week supervised exercise intervention on QoL, pain and lumbopelvic impairments in women with endometriosis not responsive to regular therapy	Randomised controlled trial<br><br>**Recruitment:** Women recruited from 2 public university hospitals in Spain<br><br>**Intervention & allocation:** Participants were randomly allocated into either the multi-modal 9-week supervised exercise program or waitlist control<br><br>**Outcome measures:** QoL measured using the EHP-30. PPT, NRS for pain intensity and PCS were also recorded. Lumbopelvic	Women with endometriosis<br><br>Intervention group n=16<br>Control group n= 15<br>4x drop out<br><br>**Lifestyle Pillar** – Physical activity	The intervention was the 'Physio-EndEA' program which is a 9 week multimodal supervised tailored program.<br><br>Week 1 involved individual session for motor control training by rehabilitative ultrasound imaging and stabiliser pressure biofeedback. The following 8 weeks involved twice weekly 90min sessions focusing on aerobic,	**Pain:** There was close-to-significant improvement in current pelvic pain (-1.63, P=0.060) and dyschezia (-1.5, P=0.970) on NRS between intervention and control post intervention however this was not found at 1 years follow up.<br><br>A significan	"Physio-EndEA" program may improve pelvic pain and dyschezia immediately following the program and dyspareunia long term.

			stability, muscle architecture of the abdominal wall and multifidus will also be measured.<br><br>**Time of outcome assessment:** Baseline, post intervention and 1 year		resistance, stretching and motor control exercises which were adapted to the physical capacity of each individual. Duration and intensity of both aerobic and resistance exercises increased over time and were individualised to each participant.<br><br>Activity levels following intervention until the 1 year follow up were not recorded.	t improvement was found in dyspareunia at 1 year follow up (Cohen's d=0.81)<br><br>**Other outcomes:** There was significant improvement in global health status, emotional well-being and pain in the intervention group at T1 which was maintained at follow up (T2) compared to the control group when	

						evaluating the EHP-30.<br><br>There was a significant improvement on the pain catastrophizing scale post intervention and at 1 year.<br><br>Pressure pain thresholds improved in both pelvic and distal nociceptive sites in the intervention group. Improvements in trunk strength and lumbopelvic stability were also observed.	
Bi &	China	To	Retrospective	Women with	The	**Pain:** At 5	There

Xie, 2018		evaluate the effect of neuromuscular electrical stimulation (NMES) for the treatment of endometriosis associated pain	study<br><br>**Intervention & allocation:** Participants divided into two groups – treatment group who underwent NMES and waitlist control<br><br>**Outcome measures:** The primary outcome is pain measured by NRS and ESSS – which required participants to rate dysmenorrhoea, dyspareunia and non-mensural pain.<br><br>The secondary outcome was quality of life utilising the SF-36.<br><br>**Timing of outcome assessment:** Baseline, 5 weeks and 10 weeks.	histologically confirmed endometriosis<br><br>Treatment group n=83<br>Wait list control n= 71<br><br>**Lifestyle pillar –** Self management	treatment group undertook NMES therapy for 30mins, 3x weekly for 10 weeks.<br><br>The gel pads for the NMES were applied with bilateral acupoints of Sanyinjiao (above the medial malleolus), Zhongji (medial lower abdomen, 4cm below umbilicus) and guanyuan (3cm below umbilicus)	weeks, there was no significant difference in pain measured by the NRS. After 10 weeks there was statistically significant improvement. NRS dropped by -2.9 (-3.7, -1.8) in the intervention group vs -0.6 (-1.0, -0.3) in the control group with a p=0.02.<br><br>**Other outcomes:**<br>At 5 weeks, there was no significan	was improvement in pain following 10 weeks of NEMS

						t difference in ESSS or quality of life measured by the SF-36.<br><br>After 10 weeks there was statistically significant improvement in all outcome measures in the intervention group compared with control.	
Breton et al., 2025	France	To measure the impact of a digital health program on the symptoms and quality of life levels of women with endometri	Cohort study Pilot study<br><br>**Recruitment:** Intervention group were recruited through employer health insurance of individual direct access. The control	Women with a diagnosis of endometriosis (clinically, imagery based or surgically)<br><br>Intervention n= 146 (however only 92 were included in the study as women who tested less than 50% of the	The digital tool (*The School of Endo*) was developed using a cognitive behavioural therapy approach (CBT) based on the endometriosis health	**Pain:** Actively following the digital program for 3 months was associated with a significant improve	Following the endometriosis specific digital health program is associated with a significant reduction

		osis	group were recruited through social media and mailing campaigns.<br><br>**Intervention & allocation:** Participation in *The School of* Endo digital tool for 3 months<br><br>**Outcome measures:** A NRS from 1-11 was used for the level of overall pain, anxiety, depression, dysmenorrhea, dyspareunia, dyschezia, dysuria, chronic pelvic pain, gastrointestinal disorders, chronic fatigue, neuropathic pain, and endo belly. The EHP-5 was also recorded.<br><br>**Time of outcome assessment:**	program content were excluded) Control group n=149<br><br>**Lifestyle Pillars –** Nutrition, physical activity, mind-body, social connection	profile (EHP) items. It focused on 5 non-pharmacological interventions of disease education (including pain mechanism), diet, adapted physical activity, well-being and mental-health and sexual health. There was a range of content including videos, exercises, written content, live sessions, quizzes and a community-based platform.<br><br>The dietary section covered good mealtime habits, why & how to make dietary changes and the role of	ment in neuropathic pain (41% vs 23.1% p=0.02), and endo belly (41% vs 24.5% p=0.03) perception among program participants when compared to the control group<br><br>**Other outcomes:** Actively following the digital program for 3 months was associated with a significant improvement in global symptom burden (20% vs	neuropathic pain compared to control group.

			Baseline and 3 months		diet in inflammation & digestive symptoms. There was also a dedicated section on anti-inflammatory foods.<br><br>The physical activity section covered ways to stay active (including a weekly 30min yoga and Pilates session). There were also ventral breathing exercises.<br><br>The mental health section focused on education, emotional regulation and relaxation practices.<br><br>The intimacy section featured physiotherap	6.1% p=0.003), anxiety (improving in 42% vs 26.5% p=0.002) & depression (deteriorating in 10% vs 27.9% p=0.003).<br><br>Active program participants also showed an improvement in their QoL at 3 months and significant improvement in knowledge on endometriosis.	

					y exercises, sex therapy tools, and advice to rediscover pleasure and self-confidence in sexual relations.<br><br>The intervention group were advised to follow this program for 3 months (with content delivered weekly).<br><br>The digital health program was developed after conducting 120 semi-structured interviews and >100 web-based questionnaires with patients and health professionals to understand the needs of women with		

					endometriosis. 12 endometriosis experts were involved in the construction of the program.		
Cirillo et al., 2023	Italy	To evaluate the role of the Mediterranean diet on pain perception in endometriosis and their relationship with oxidative stress	Prospective study<br><br>**Recruitment:** Women were recruited from Endometriosis Centre of Careggi University Hospital, Florence<br><br>**Intervention & allocation:** All participants were advised to follow a Mediterranean diet<br><br>**Outcome measures:** Pain intensity utilising VAS for dyspareunia, non-menstural pelvic pain, dysuria and dyschezia	Women with endometriosis, diagnosed via imaging (USS or MRI) or laparoscopically<br><br>N=35<br>- 35 completed 3 months, 26 completed 6 months<br><br>**Lifestyle Pillar –** Nutrition	Women underwent baseline assessment of current nutritional habits. They then completed a 10 day food diary and from this a personalised tailored nutrition Mediterranean Diet plan was developed and agreed on.<br><br>Women then followed this diet for 6 months.	**Pain:** At 3 months, patients reduced pain in terms of dyspareunia (p = 0.04), nonmenstrual pelvic pain (p = 0.06), dysuria (p = 0.04), and dyschezia (p < 0.001). Dyspareunia (p = 0.002) and dyschezia (p < 0.001) where even more significantly	Significant improvement in dyspareunia, non-mensural pelvic pain, dysuria and dyschezia when adhering to a mediterranean diet.

			The mediterranean diet adherence score was also measured throughout.<br><br>Vitamin profiles and oxidative stress markers, such as lipid peroxidation) were also recorded.<br><br>These secondary outcomes of pain and oxidative stress came out of the original study which looked at atherosclerosis & endometriosis & the role of diet.<br><br>**Timing of outcome assessment:** baseline (T0), after 3 months (T1) and 6 months (T2) of mediterranean diet.			reduced after 6 months.<br><br>Improvement may be due to anti-inflammatory aspects of the diet – rich In omega 3 fatty acids and extra virgin olive oil	

De Hoyos et al., 2023	Spain	To adapt and test the efficacy of an environmental enrichment (EE) intervention of pelvic pain, mental health, perceived stress, quality of life and systemic inflammation in endometriosis patients	Randomised controlled trial A Pilot study.<br><br>**Recruitment:** Participants were recruited utilising social media.<br><br>**Intervention& allocation:** six EE modules and had a WhatsApp chat to communicate between sessions. Participants were randomised into either the treatment arm or wait-list control.<br><br>**Outcome measures:** 1-10 NRS for pain, EHP-30, PSS, GAD -7, PHQ-8, PSS and PCS. Saliva samples were also collected.<br><br>**Time of outcome assessment:** Baseline and 3 months	Women with a surgical diagnosis of endometriosis with ongoing symptom.<br><br>Intervention group n=29 Waitlist control n=27<br><br>**Lifestyle Pillars –** Mind-body, social connection	EE is a psychosocial mind-body intervention consistent of social and cognitive stimulations (social support, novelty, and exposure to open spaces).<br><br>This EE intervention comprised of six fortnightly modules which involved the intervention undertaking a support group meeting, followed by a novel stress-management activity in an open space. Activities included yoga, yogic breathing, mindfulness, aromatherapy, art therapy, drama therapy and dance	**Pain:** The intervention and control groups showed similar and not statistically significant change in global pain impact scores at baseline and end over intervention.<br><br>**Other outcomes:** There was a statistically significant improvement in GAD-7 in the intervention group at the end of the interventi	EE did not result in any significant improvement in pain.

					therapy while locations included the beach, lake, garden, hot springs and countryside.<br><br>Those in the waitlist control were invited to participate in an online seminar about endometriosis. Both groups could continue to receive standard gynaecological or psychological therapy as needed.	on (p =0.006) and 3 months after (p<0.0001). Similarly, there was improvement in depressive symptoms in the PHQ-8 with lower levels in the intervention group (p=0.014 end of intervention, p=0.006 3 months post).<br><br>There was a significant decrease in PSS score at 3 month post intervention (p=0.046)	

						. <br>There was no statistically significant changes in salivary cortisol. <br>Intervention was well received by participants.	
Goncalves et al., 2016	Brazil	To evaluate the impact of a Hatha Yoga program on chronic pain, menstrual patterns and QoL in women with endometriosis	Randomised controlled trial <br>**Recruitment:** Department of Obstetrics & gynaecology, University of Campinas Medical School <br>**Intervention & allocation:** Twice weekly yoga vs control group. Control group remained on standard therapy +/- one individual physical therapy session a	Women with a confirmed diagnosis of endometriosis and chronic pain (VAS >4) despite conventional therapy <br>Treatment group – n=28 Control group – n=12 <br>**Lifestyle Pillar –** mind-body	Participants in the intervention group undertook 2 hour yoga sessions twice weekly for 8 weeks. <br>Each session was lead by the same qualified yoga instructor. Each session involved 30mins conversation & interaction between participants, 10 mins of	**Pain:** EHP-30 pain domain improved from a mean of 60.80 to 32.39 in the intervention group compared to a change of 58.71 to 55.05 in the control group. This is statistically significan	Significant improvement in pain on the EHP-30 & daily pain score in intervention group

			week.<br>Random computer generation in 3:1 ratio.<br><br>**Outcome measures:** EHP-30, mensural and daily pain scale (on a VAS)<br><br>**Timing of outcome assessment:** initially and after the 8 week program		relaxation with pranayamas and body awareness, 60mins of asanas (hatha yoga postures), 10 mins meditation/breathing techniques/chanted mantras and 10 mins of feedback. The same asanas where used each session	t p=0.0046.<br><br>The pain VAS score showed a statistically significant improvement in the intervention group (p=0.0007).<br><br>**Other outcomes:** No significant change in menstrual patterns. Improvement in QoL were statistically significant for importance (p=0.0006), well-being (p=0.000	

						9) and image (p=0.0087).	
Goncalves et al., 2016	Brazil	To understand the meaning women with pain-associated endometriosis attribute to yoga practice	Qualitative study conducted simultaneously with a randomised controlled trial (Goncalves et al. 2017)<br><br>**Intervention & allocation:** Twice weekly yoga session for 8 weeks<br><br>**Outcome measures:** a single semi-structured interview.<br><br>**Timing of outcome assessment:** Following completion of the 8 week program	The original RCT contained 40 participants – 28 assigned to the yoga group and 12 to the non-intervention group.<br><br>15 women who had completed the 8-week yoga program participated in this qualitative study.<br><br>**Lifestyle Pillar –** mind-body	Each yoga session was led by a physical therapist/yoga instructor. It consisted of 30mins interactives between participants followed by relaxation, diaphragmatic breathing exercises, hatha yoga postures and psychophysical relaxation. Sessions occurred twice weekly for 8 weeks.<br><br>15 intervention participants undertook a semi-structured interview at the end of the 8 week program.	**Pain:** All participants reported that yoga was beneficial to control pelvic pain.<br><br>**Other outcomes:** Women identified a relationship between pain management and breathing techniques.<br><br>They created ties among themselves, suggesting that the yoga group	Yoga was beneficial to control pelvic pain.

						allowed for psychosocial support.	
Hansen et al., 2017	Demark	To evaluate the long-term effects of a mindfulness based psychological intervention on chronic pain and QoL in endometriosis patients	Follow up of prospective observational pilot study (Kold et al, 2012)<br><br>The 10 women involved in the original study were re-contacted to participate in this follow up. All agreed.<br><br>**Outcome measures:** EHP-30 and SF-36.<br>They were also questioned about their ongoing use of the mindfulness based interventions, overall experience of QoL and general pain in a 5 point leichhardt scale<br><br>**Timing of**	As above in Kold et al, 2012	As above in Kold et al, 2012<br><br>During the 6 years since the original study, one women had entered natural menopause, three had had endometriosis lesions removed and two had had a hysterectomy.<br><br>9 out of 10 still used the mindfulness-techniques and other mental techniques learnt during the intervention. Body-scanning and breathing techniques were the	**Pain:**<br>8 out of 10 patients experienced a better or much improved pain level compared to prior the intervention.<br><br>**Other domains:**<br>When comparing data from the 12 months follow-up with data from the six year follow-up, results showed no significant differences in	Improvements seen at 12 months following a bMBI were maintained 6 years following intervention.

			**outcome assessment:** 6 years following original intervention		most utilised.	mean scores on all scales of the EHP-30 and almost all scales of the SF-36 scale scores (and thus improvements seen at 12 months were sustained)	
Hansen et al., 2023	Denmark	To evaluate the effect of psychological interventions on chronic pelvic pain and quality of life in women with endometriosis.	Three armed parallel, multi-centre randomized controlled trial<br><br>**Recruitment:** three specialised outpatient clinics for endometriosis in Denmark and from the Danish endometriosis patient's association.<br><br>**Intervention & allocation:**	Women with endometriosis diagnosed by surgery or MRI who have ongoing pelvic pain >5 on a NRS.<br><br>MyENDO n=19<br>Non-specific n=19<br>Wait list control n=16<br><br>**Lifestyle Pillar –** Mind-body	MyEndo is 10-week program combining mindfulness based stress reduction (MBSR) and acceptance and commitment therapy (ACT) techniques. It was delivered by a 3-hour weekly group session with patients provided education about	**Pain:** Compared to control group, the psychological intervention (MY-ENDO or non-specific) did not significantly reduce chronic pelvic pain (Cohen's	Compared to control group, the psychological intervention (MY-ENDO or non-specific) did not significantly reduce chronic pelvic pain.

			Participants were randomised to one of three groups<br>- Specific mindfulness and acceptance based psychological intervention (MY-ENDO)<br>- Non-specific psychological intervention<br>- Waitlist control<br><br>**Outcome measures:** Primary		endometriosis, group therapy on their experiences and coping mechanisms and a variety of mindfulness and yoga exercises. Patients were also encouraged to practice 30-45 mins of mindfulness meditation and yoga at home 5-7 days a week.<br><br>The non-specific psychological intervention group underwent 10 weekly sessions which involved relaxation while listening to soft, relaxation music and guided physical training	d 0.180).<br><br>**Other outcomes:** Improve the QoL domains of 'control and powerlessness', 'emotional wellbeing' and 'social support' along with improvement in the symptoms of dyschezia and constipation.	

			outcome was pelvic pain intensity/unpleasantness measured by a 0-10 point NRS. Secondary outcomes included endometriosis related QoL, workability, pain acceptance, and endometriosis-related symptoms. Participants were also encouraged to keep a 12 week pain diary.<br><br>**Timing of outcome assessment:** Questionnaires were completed prior to intervention and again post the 12 weeks of treatment .		(warm up, muscle training and stretching). They were also encouraged to practice relaxation and physical training at home 5-7 days a week.		
Kold et al., 2012	Denmark	To evaluate the effect and	Prospective observational study<br>Pilot study	Women with endometriosis who are still experiencing	10 x1.5hr-session intervention – which	**Pain:** The EHP-30 domain of pain	The EHP-30 domain of pain

		feasibility of mindfulness based psychological interventions on chronic pain management secondary to endometriosis	**Recruitment:** Aarhus University Hospital Denmark.<br><br>**Intervention & allocation:** All participants undertook 10-weekly mindfulness based intervention sessions (5 individual and 5 group)<br><br>**Outcome measures:** self-reported questionnaires of general health status (SF-36) and EHP-30.<br><br>**Timing of outcome assessment:** pre-intervention, post intervention and again at 6mth and 12mths post intervention.	pronounced pain despite standard therapy.<br><br>N=10<br><br>**Lifestyle Pillar –** Mind-body	included 5 individual sessions and 5 group sessions<br><br>Group sessions involved mindfulness techniques (body-scan, sensory training, breathing techniques and bio-feedback), psychoeducation (grief processes, emotions associated with adapting to chronic pain, stress & pain, works issues, health habits including food and exercise, social network and mind-body) and group counselling. Individual sessions involved mindfulness techniques,	improved significantly from 52.53 pre-intervention to 33.18 post intervention, 31.59 at 6mths and 28.12 at 12 months (p=0.003).<br><br>**Other domains:** All domains of the EHP-30 improved with the intervention and remained improved at 6 and 12 month follow up (except for self-image which improvement was temporary).	improved significantly following a bMBI program.

					counselling/supportive therapy and one session involving partner.<br><br>Participants were also required to do at-home mindfulness	The SF-36 showed significant improvement in all 8 scales.	
Lutfi et al., 2023	Australia	Evaluate the immediate impact of a single session of 'supervised' telehealth-delivered exercise compared to 'self-managed' virtual reality (VR) delivered exercise on pelvic pain associated with endometriosis	Randomised controlled trial Pilot study<br><br>**Recruitment:** general practices, university newsletters, study websites and other social media.<br><br>**Intervention & allocation:** Participants were randomised into a single session of 'supervised' telehealth-delivered exercise or 'self-managed' virtual reality (VR) or control group<br><br>**Outcome**	Women >18 with endometriosis<br><br>3x groups:<br>VR delivered exercises (n=8)<br>Telehealth delivered exercise (n=8)<br>Control (n=6)<br><br>**Lifestyle intervention –** physical activity	Telehealth-delivered exercise intervention was a 1 hour supervised session which included cardiorespiratory exercise (intervals, with 4min exercise bouts followed by 2 mins rest repeated 3 times), stretching and specific stabilising exercises of muscles within the lumbopelvic area. The cardiorespiratory intensity was prescribed	**Pain:** There was no significant pain VAS score change between the groups following the training intervention.<br><br>There was an increase in pelvic pain scored in baseline across the three groups, however both intervention	There was no significant decrease in pain following a VR or supervised telehealth exercise program.<br><br>However, the pain in both intervention groups increased less than the control and thus either may have the capacity

			**measures:** Acute pelvic pain was evaluated using a 100mm VAS<br><br>**Time of outcome assessment:** baseline and 48hr following a single bout of exercise (VR or telehealth) or no intervention (control)		according to ventilation thresholds and HR.<br><br>The VR exercise intervention involved participants completing a 1 hour unsupervised session. It included a 10min VR pain-distraction experience followed by 50mins of exercise using one of the following applications (based on individual participants preferences) – Dance Central, Beat Saber, The Thrill of the Fight, Space Pirate Trainer, Fruit Ninja, OhShape, Racket NX, Table Tennis VR, Rachek Fury, Swords	groups showed a lower magnitude increase in pain score.<br><br>VR increase +9 +/- 24mm, telehealth +10 +/- 12mm, control +16 +/- 12mm.	to elicit a hypoalgesia effect on pelvic pain.

					of Gargantua, Box VR, Superhot VR, VZ Fit Play and VZFit explorer)		
Marziali et al., 2012	Italy	To evaluate the effectiveness of a gluten free diet in women with endometriosis related chronic pelvic pain	Retrospective observational case series.<br><br>**Recruitment:** Office of Endometriosis Centre and educated on how to follow a complete gluten free diet.<br><br>**Intervention & allocation:** Gluten free diet<br><br>**Outcome measures:** a 0-10 VAS evaluating dysmenorrhoea, non-menstrual pelvic pain and dyspareunia<br><br>**Time of outcome assessment:** At enrolment and 12 months post intervention.	Women with endometriosis with moderate-to-severe painful symptoms.<br><br>Those with coeliac or other gastro-intestinal disease were excluded.<br><br>Overall, 207 patients completed the study.<br><br>330 recruited initially, only 207 who showed improvement in symptoms after 2 weeks completed the 6 month protocol<br><br>**Lifestyle Pillar –** Nutrition	Women were educated to follow a complete gluten free diet<br><br>Participants were asked to keep a diary of daily dietary intake & painful symptoms throughout	**Pain:** At 12 months follow up, 75% of patients reported statistically significant improvement in painful symptoms (P<0.005).<br><br>**Other outcomes:** There was a significant increase in score of all domains of physical functioning, general health	Following a gluten free diet for 12 months resulted in a statistically significant improvement in painful symptoms in 75% of participants.

						perception, vitality, social functioning and mental health.	
Merlot et al., 2023	France	Assess the effects of repeated at-home virtual reality (VR) on pelvic pain due to endometriosis on the five most painful consecutive days of the month	Randomized controlled trial Double-blinded<br><br>**Intervention & allocation:** Participants randomised into intervention or sham program.<br><br>VR headsets twice daily for at least 2 days, and up to 5 days, starting on the first day of their painful period. The intervention group utilised a VR solution (Endocare) while the control group underwent a sham program.<br><br>**Outcome measures:** Pain perception was measured	Women with endometriosis and/or adenomyosis with at least 2 consecutive days each month of moderate-severe pelvic pain.<br><br>120 patients were initially recruited, however only 102 were included in the study analysis. 36 had a diagnosis of andenomyosis (16 in control, 20 in intervention)<br><br>Intervention group n=51<br>Sham group n=51<br><br>**Lifestyle Pillar –** Mind-body, other	Endocare is a digital therapeutic device which consists of visual and auditory therapeutic procedures administered via a VR headset. Each session lasts for 20mins and consists of components such as binaural beats, verbal hypnotic injunction, nature-based sounds, distraction of attention and bilateral alternative stimulations. The control group utilised a sham VR program which consisted of a	**Pain:** Compared to pre-treatment pain intensity levels, pain intensity reduction reached 51.6% at 120mins and 51.2% at 180mins on D2 in the Endocare group vs 21.2% and 23.9% in the control group. Pain intensity reduction was also significantly higher in the Endocare Group on	Pain intensity reduction was significantly higher in the Endocare Group on D1, 2 and 3 compared to control. There was no difference observed in the groups on D4 and D5.

			using a NRS (0-10).<br>General pain, stress, fatigue, medication intake and QoL were also recorded daily utilising a VAS, EHP-5, and PCS.<br><br>**Time of outcome assessment:** before and then 60, 120 and 180 mins after each treatment administration.		20-minute audio-video composition.<br><br>Patients were encouraged to use the VR device twice daily for at least two (and up to 5) of the most painful consecutive days of their cycle. There was significant decrease in uptake on D3, 4 and 5.	D3. There was no difference observed in the groups on D4 and D5.<br><br>In the first 2 days, patients with Endocare experienced significantly higher pain relief than patients from the control group (except on D1 60mins).<br><br>There was no significant difference in medication use between the two groups.	

						**Other outcomes:**<br>There was no significant difference in effect between intervention and control at wakeup and bedtime (there was a reduction in pain intensity ad bedtime, however this was similar between the intervention control group)<br><br>There was no significant difference in fatigue	

						and stress between the two groups (both had reduction in fatigue and stress from D1 to D5).	
Miazga et al., 2024	Canada	Assess the effectiveness of a virtual mindfulness-based stress reduction (MBSR) program to improve QoL and pain in people with endometriosis	Multiple-method, before & after study design<br><br>**Recruitment:** Recruited from a Canadian outpatient gynaecology clinic.<br><br>**Intervention & allocation:** All participants undertook a virtual mindfulness based stress reduction program (online via Zoom)<br><br>**Outcome measures:** VAS were used to assess dysmenorrhoea, dyspareunia	Women with a surgical or clinical diagnosis of endometriosis.<br><br>N=15<br><br>**Lifestyle pillar –** mind-body, social-connection	The virtual MBSR program was run by an experienced social worker in an 8 week period over zoom. Each session was 2.5 hours long. It incorporated core components of meditation, education on mindfulness practices and emotional regulation training along with specific endometriosis education and information regarding intimate	**Pain:** There was no significant change in pain scores or medication use.<br><br>**Other outcomes:** Statistically significant increase in control and powerlessness (p=0.012), emotional well-being (p=0.048), social support (p=0.030)	A virtual MBSR did not improve pain or medication use in women with endometriosis.

			and chronic pelvic pain.<br><br>The EHP-30 was also assessed along with a pain-medication use questionnaire.<br><br>A focus group was also help at the completion of the program.<br><br>**Timing of outcome assessment:** Before intervention, then immediately following the 8-week MBSR program.		relationships and fertility. Participants were also given home exercises to practice between sessions.<br><br>67% (10/15) completed the MBSR program	and self-image (p=0.014) follow the MBSR program.<br><br>The qualitative component identified that participants found the sense of community, education and application of mindfulness tools when approach pain the most beneficial components of the intervention. They found therapeutic benefits from sharing their	

						experiences with the disease.	
Mira et al., 2015	Brazil	To evaluate the effectiveness of TENS as a complementary treatment in chronic pelvic pain and deep dyspareunia in women with deep endometriosis	Randomised controlled trial<br><br>**Intervention & allocation:** Participants were randomised into two different treatment arms:<br>Group 1 was acupuncture-like TENS (frequency 8Hz, pulse duration 250us) and group 2 self-applied TENS (frequency 85Hz, pulse duration 75us).<br><br>**Outcome measures:** VAS for pain – chronic pelvic pain, dyschezia, dysuria and dysmenorrhoea – and deep dyspareunia was evaluated using the DDS.	Women with deep endometriosis (with endometriosis in the cul-de-sac and/or intestinal loop) undergoing hormonal treatment with ongoing persistent pelvic pain and/or dyspareunia.<br><br>Group 1 – n=11<br>Group 2 – n= 11<br><br>**Lifestyle pillar –** Self management	Two treatment arms<br>Group 1 was acupuncture-like TENS (frequency 8Hz, pulse duration 250us) and group 2 self-applied TENS (frequency 85Hz, pulse duration 75us).<br><br>The TENS was applied in the S3/4 region in both groups.<br><br>Acupuncture-like TENS occurred in 30min, weekly sessions for 8 weeks. Self-applied TENS occurred for 20mins twice daily for 8 weeks.	**Pain:** For all women, TENS provided symptomatic pain relief, with significant differences before and after chronic pelvic pain treatment ($p < .0001$), deep dyspareunia ($p = .001$) and dyschezia ($p = .001$).<br><br>There was no significant improvement in dysmenorrhoea or dysuria.	Both acupuncture-like TENS and self-applied TENS demonstrated effectiveness in chronic pelvic pain, deep dyspareunia and dyschezia in women with deep endometriosis. There was no significant improvement in dysmenorrhoea or dysuria.

			The EHP-30 was also recorded<br><br>**Timing of outcome assessment:** All outcomes were measured before and after treatment (8 weeks).			**Other outcomes:** The EHP-30 demonstrated improvement in pain, control & powerlessness, emotional wellbeing, social support and self-image domains.<br><br>There was no significant difference between the two treatment arms.	
Mira et al., 2020	Brazil	Evaluate the impact of self-applied electrotherapy treatment for pain control over	Multi-centre randomized controlled trial.<br><br>**Intervention& allocation:** Participants were randomised to	Women with deep infiltrating endometriosis diagnosed via USS or MRI who experienced ongoing pelvic pain despite hormonal treatment.	The intervention group was treated with self-applied transcutaneous electrical nerve stimulation device	**Pain:** Chronic pelvic pain improved in the intervention group with VAS decrease	The use of self-applied TENS has benefits in the relief of chronic pelvic pain with

		standard hormonal treatment for deep infiltrating endometriosis	either the intervention or the control group.<br><br>The intervention group received the TENS therapy while the control group did not. Both groups continued to receive standard hormonal therapy.<br><br>**Outcome measures:** Primary outcome was chronic pelvic pain utilising a VAS (for chronic pelvic pain, dyschezia, dysmenorrhoea) and deep dyspareunia (via DDS). Secondary outcomes were QoL (EHP-30) and sexual function (FSFI).<br><br>All participants also undertook	Intervention (hormonal therapy + electrotherapy) n=53<br>Control (standard hormonal therapy) n=48<br><br>**Lifestyle pillar –** Other, self-management	(TENS). Participants were advised to choose an intensity which was 'strong but comfortable'. The device frequency was 85Hz and pulse duration 75us.<br><br>The device was used in the parasacral region (S3/S4 position) for 20mins twice daily for 8 weeks.	from 7.11 +/- 2.4 to 4.55 +/- 3.8, p<0.001, 36%) which was not observed in the control group (VAS change from 7.33 +/- 2.09 to 7.06 +/-2.33, p=0.554, 3.68%). Deep dyspareunia also decreased 32.67% compared with 13.84%. The number of days each week of pelvic pain also improved from 3.27 to 2.22 (p=0.028, 32.11% decrease) in the	a decrease in chronic pain and deep dyspareunia along with few pelvic pain days and decreased medication use in women with endometriosis.

			a symptom diary throughout.<br><br>**Timing of outcome assessment:** before treatment and after 8 weeks of treatment.			intervention group.<br><br>The intervention group too 7 days of medication, compared to 18 days in the control group.<br><br>**Other outcomes:** There was an improvement in QoL across all domains of the EHP-30 in the intervention group and suggested improvement in sexual function.	
Moreira et al., 2022	Brazil	To investigate the effects	Randomised controlled trial.	Women with deep endometriosis	The bMBI program was delivered in 2	**Pain:** bMBI significan	bMBI significantly

		of a brief Mindfulness Based intervention (bMBI) in women with deep endometriosis on general pelvic pain, and specific pain descriptor namely dysuria, dyschezia, dyspareunia and dysmenorrhoea	Pilot study.<br><br>**Recruitment:** Endometriosis Outpatient Clinic of the Pedro Ernesto University Hospital, Brazil<br><br>**Intervention & allocation:** Participants randomised into standard medical treatment plus bMBI and only standardised medical treatment as a control.<br><br>The intervention group underwent guided bMBI training which was delivered by a mindfulness-based stress reduction teacher who had extensive experience in chronic pain.<br><br>**Outcome measures:** The 11-item	with ongoing pain despite standard medical care.<br><br>Total of 63 participants:<br>- bMBI (n=31)<br>- Control (n=32)<br><br>**Lifestyle Pillar –** Mind-body	cycles with 15 & 16 women in each class. There were 4 on-site mindfulness classes which ran for around 90min each. This was followed by a 3 week follow up period during which time participants received weekly online mindfulness exercises and were encouraged to maintain mindfulness training.<br><br>Each bMBI class was divided into four segments – psychoeducation, mindfulness based meditation practice, guided enquiry of participants	tly improved the primary outcomes of pelvic pain, dyschezia, dysmenorrhoea and pain unpleasantness at post treatment compared to control (NNT ranging from 5 to -9 and Cohens = 0.23).<br><br>**Other outcomes:** bMBI also improved the SF-36 vitality (NNT 2.1, Cohen's = 0.22) and SF-36 mental health (NNT 3.5 and	improved the primary outcomes of pelvic pain, dyschezia, dysmenorrhoea and pain unpleasantness at post treatment compared to control.

			Pain NRS was utilised for pain, the 10-item Brazilian version of the PSS was utilised for stress and health related QoL was measured utilising the SF-36.<br><br>**Time of outcome assessment:** baseline (T0), 5 weeks (T1) and 8 weeks (T3) from intervention initiation.		experience and home exercise instructions. The psychoeducation included reconceptualising pain, mindfulness attitudes, stress response, interactions between thoughts, emotions, body sensations and their role in stress, pain and well-being. Formal meditation practice included mindful movement, body scan, mindfulness of breath, walking, meditation, and mindfulness of breath, body and thoughts.<br><br>Participants were encouraged	Cohen's = 0.34) compared to control. There was only marginal non-significant effect of the bMBI on stress perception reduction.	

					to completed 20-30mins of formal meditation daily (using recordings) and incorporate mindfulness of attention to at least one activity, and 3 mins of body-breath meditation 3x daily.<br><br>The control group continued with standard medical care utilising hormonal therapy and analgesics.		
Ott et al., 2012	Austria	To explore whether a diet which closely follows Mediterranean nutritional recommendations affect endometriosis associated pain	Prospective, experimental observational study<br><br>**Recruitment:** Medical University Vienna, hospital.<br><br>**Intervention & allocation:** All participants were advised to follow a	Women with endometriosis confirmed on laparoscopy with ongoing pain.<br><br>Participants could be not be taking oestrogen suppressive drugs such as the oral contraceptive, danazole or GnRH analogues in the 6 months	Participants were surveyed on the telephone twice – initially focused on their current dietary habits and endometriosis symptoms (such as dysmenorrhoea, dyschezia,	**Pain:** The intention to treat analysis demonstrated a significant improvement in pain based on NRS (4.2±2.5 to	Five months following the mediterranean diet led to a significant improvement in pain, dysmenorrhoea, dyspareu

			mediterranean style diet for 5 months.<br><br>**Outcome measures:** Change in subjective pain sensations, measured via a NRS.<br><br>**Timing of outcome assessment:** Prior to intervention and after 5 months of adherence to the mediterranean diet	prior to dietary therapy. Women could take NSAIDs if required.<br><br>N=68<br><br>**Lifestyle Pillar –** nutrition	dyspareunia and dysuria) and then again after the 5 months of dietary intervention.<br><br>A mediterranean style diet was explained and recommended to patients - fresh vegetables and fruit, white meat, fish rich in fat 3x weekly, soy products, wholemeal products, foods rich in magnesium and cold-pressed oils. Sugary drinks, red meat, sweets and animal fats were to be avoided.<br><br>The diet was to be adhered to for 5 months.<br><br>43% of	2.5±2.4; p<0.01).<br><br>Patients also experienced significant improvement in dysmenorrhoea, dyspareunia and dyschezia (P<0.01).	nia and dyschezia

					patients (63.2%) adhered to the diet.		
Ravins et al., 2023	Israel	To examine the effect of practicing endometriosis yoga on the stress and QoL of women diagnosed with endometriosis	AB (baseline then intervention) study design Pilot study<br><br>**Intervention & allocation:** all participants completed 8 weeks of conventional therapy followed by 8 weeks of twice weekly 90-minute endometriosis yoga classes.<br><br>**Outcome measures:** EHP-30, NRS for pain and intensity of bleeding during last menstrual period.<br><br>**Time of outcome assessment:** Prior to conservative treatment (T1), following the two	Women with a diagnosis of endometriosis confirmed by a gynaecologist with ongoing pelvic or back pain.<br><br>52 women were included in the study. 42 women completed the whole 4 month study course.<br><br>**Lifestyle Pillar –** mind-body, physical activity, social connection	Endometriosis yoga emerged from 'yoga Nashit' which was original established specifically for the anatomy and physiology of a woman's body. The endometriosis yoga method teaches practitioners to develop a different response to pain through different means such as empathetic attention to painful areas, relaxation postures, breathing techniques, and gentle movement sequences. Women are encouraged to observe	**Pain:** NPRS for pelvic pain improved by 0.97 +/- 0.32 (p=0.01) between T2 and T3. There was no difference between T1 and T2.<br><br>**Other outcomes:** The EHP-30 score improved by 10.43 +/- 2.59 (P=0.001).<br>When looking at the EHP-30, the variables of pain, control & powerlessness, emotiona	8 weeks of endometriosis yoga significantly improved pelvic pain in women with endometriosis

			months of conservative treatment (T2) and following the two months of yoga (T3).		and share their emotions so that they can receive support from the facilitator and other members of the group.<br><br>The study took place during 2021 and thus the twice weekly yoga sessions occurred online. The first lesion each week was a group class on zoom, while the second was a recording. There were 8-9 women in each group with one qualified yoga instructor.<br><br>Each class started with 15 minutes of conversation (including breathing techniques	l well-being, social support, work and intercourse improved the most.<br><br>NPRS bleeding intensity improved by 0.44 +/- 0.15 (p=0.019) between T2 and T3. There was no difference between T1 and T2.	

					and emotional & motivational support) followed by 10 mins physical & psychological relaxation, 10 mins warm up, 40 mins of asanas, 20 mins restorative asanas, 5 mins intuitive writing and 5 mins relaxation.		
Rohloff et al., 2024	Germa ny	To examine whether the "Endo-App" improves QoL in women with endometri osis (and whether a RCT should take place)	Observational pilot study<br><br>**Recruitment:** self-help groups, advertising on social media and posting in endometriosis related groups.<br><br>**Intervention & allocation:** All participants were advised to use the Endo-App<br><br>**Outcome measures:** Outcome measures	Women with endometriosis<br><br>N=106<br><br>n=64 reported using the app and n=42 didn't use the app in the 2 week period<br><br>**Lifestyle Pillar** – mixed	The Endo-App is designed to support the multi-modal therapy of endometriosi s. It provides evidence based and guidelines-compliant content with features such as an endometriosi s diary, exercise guides, nutrition advice, educational articles and	**Pain:** The QoL domain of pain improved by 9.23 points on the EHP-30 with a Cohen's d of 0.73.<br><br>**Other outcome s:** There was a statistical ly significan t improve ment in QoL	The QoL domain of pain improved by 9.23 points on the EHP-30 with a Cohen's d of 0.73 following two weeks of use of the Endo-App.

			were QoL utilising the EHP-30 and quality of life-index. The stage of the menstrual cycle was also taken into account.<br><br>**Timing of outcome assessment:** Participants undertook an online questionnaire prior to the study, and then again after using the Endo-App for 2 weeks.		videos, psychological support, stress-reducing concepts and guidance on positive coping. There is also an emergency plan to assist in managing severe pain.	following two weeks of use of the Endo-App. The EHP-30 domains of work-life, control & helplessness and pain improved the most, while self-image only improved a small amount and was not statistically significant.	
van Haaps et al., 2023	Netherlands	To explore the influence of dietary interventions – namely low FODMAP and endometriosis diet – on	Prospective study with control group<br><br>**Recruitment:** Endometriosis Centre of the Amsterdam University Medical centre.<br><br>**Intervention &**	Women with endometriosis (diagnosed surgically or radiologically) receiving insufficient benefit from their current medical treatment<br><br>Total of 62	Dietary guidance for both diet groups was 3x 1 hour consultations and 3 short 30min consultations over a 3 month period.	**Pain:** All participants adhering to diet reported significantly less dyspareunia, dysuria, bloating	Statistically significant less dyspareunia, dysuria, bloating and tiredness in both low FODMAP

		endometriosis related pain and quality of life (QoL)	**allocation:** Participants chose between adherences to a diet (either low FODMAP or endometriosis diet) or no diet. Those in the diet group received guidance from a dietician in training for 3 months.<br><br>**Outcome measures:** pain VAS (scale 0 -10cm) for dysmenorrhoea, deep dyspareunia, chronic pelvic pain, dysuria, and bloating – along with a GIQoL questionnaire and the EHP-30. Those in the dietary intervention groups also received a VAS on adherence to their chosen diet.<br><br>**Time of outcome	participants<br>- Low FODMAP diet (n=22)<br>- Endometriosis diet (n=21)<br>- Control (n=19)<br><br>**Lifestyle Pillar:** Nutrition	Participants were provided with written materials – consisting of suitable recipes, weekly menus, grocery & practical tips for adherence.<br><br>FODMAPs are a large class of non-digestible carbohydrates found in many foods including some fruits, vegetables, honey, sweeteners, milk and dairy products. They result in increased water absorption into the GI tract and then fermentation, which contributes to symptoms such as	and tiredness after adhering to the diet for 6 months compared to their baseline (range P<0.001 to P=0.012). However, when compared to the control group, only deep dyspareunia in low FODMAP and bloating in endometriosis diet remained statistically significant.<br><br>Participants in control group reported no significan	and endometriosis diet groups at 6 months compared to their baseline.<br><br>However, compared to control group only deep dyspareunia in the low FODMAP group remained statistically significant.

			**assessment:** at the start of the study (T0), at 3 months (T1) and at 6 months (T2)		bloating, flatulence, abdominal pain and constipation. The low FODMAP diet was initially developed in the management of IBS.<br><br>The low FODMAP diet is an avoidance diet with consists of 3 phases – phase 1, eliminate all high FODMAP foods for 6 - 10 weeks; phase 2 – challenge – reintroduce one high FODMAP nutrient every 3 days to see whether exposure causes IBS symptoms; phase 3 – once all the high-FODMAP	t difference in pain scores or endometriosis symptoms.<br><br>**Other outcomes:**<br>All participants adhering to diet scored significantly better on the QoL domains of pain, powerlessness, emotional wellbeing, self-image, work life and sexual intercourse after 6 months compared to their baseline. When compare	

					foods have been challenged patients will have a personalised diet based on their tolerance.<br><br>The endometriosis diet was developed by women with endometriosis after noticing certain nutrients flared symptoms. It was standardised for this study based on a recent survey among Dutch endometriosis patients. Nutrient groups to exclude included: red meat, gluten, cow's milk, sugars, nutrients high in oestrogen, limit caffeine (to 200mg	d to the control group, the only statistically significant QoL result was in social support and medical profession.	

					daily).<br><br>The control group did not receive any dietary advice of intervention.		

FODMAP – fermentable oligosaccharides, disaccharides, monosaccharides and polyols, VAS – visual analogue scale, GIQoL – gastro-intestinal quality of life, EHP – endometriosis health profile, NRS – numeric rating scale, PSS – perceived stress cale, SF-36 – short form health survey, GAD - generalised anxiety disorder 7, PHQ – patient health questionnaire 8, PCS – pain catastrophising scale, PPT – pressure pain thresholds, FSFI – female sexual function index, DDS – deep dyspareunia scale, ESSS – endometriosis symptom severity score
